# The impact of the rural digital economy on China’s new-type urbanization

**DOI:** 10.1371/journal.pone.0321663

**Published:** 2025-04-24

**Authors:** Nan Chen

**Affiliations:** College of Economics and Management, Jilin Agricultural University, Changchun, China; Wenzhou University, CHINA

## Abstract

The Chinese government is vigorously implementing the rural revitalization strategy and accelerating the process of new-type urbanization. The rapid development of the rural digital economy has emerged as a new driving force for new-type urbanization. This study aims to explore how the rural digital economy impacts China’s new-type urbanization from direct, heterogeneous, and indirect perspectives. Using the provincial-level panel data in China from 2014 to 2022, a mixed-methods approach is employed for the empirical research. The CRITIC and Entropy TOPSIS are used to assess the comprehensive development level and temporal characteristics of the rural digital economy and new-type urbanization. Moreover, a global-local auto-correlation analysis is carried out to depict the spatial distribution of the two variables. Subsequently, a two-way fixed effects model is constructed to verify the direct impact of the rural digital economy on new-type urbanization, as well as its structural and spatial heterogeneity characteristics. Finally, an mediating effect model is established to explore the impact paths through which the rural digital economy impacts new-type urbanization. The results show that the rural digital economy has significantly promoted new-type urbanization. Specifically, rural digital infrastructure, digital transformation of agriculture, agricultural production service informatization have a significant positive effect, while the role of rural life digitization is not significant. The rural digital economy has more significant positive impact on population agglomeration and economic growth, followed by social public service, but has no significant impact on ecological environmental protection and urban-rural coordination. Additionally, the qualitative analysis identifies geographical region, poverty, demographic structure and social equality as notable influencing factors in this impact. Further mechanism analysis result indicates that the rural digital economy impacts new-type urbanization through rural human capital improvement, agricultural economic growth and rural industrial structure upgrading. This research contributes to the existing body of knowledge by providing the practical path of rural development to promote new-type urbanization in the context of the digital economy, also clarifies the weak points and key links in this process. It also highlights the need for further research into the institutional factors that influence this relationship to enhances the policy applicability.

## Introduction

### Background and connotation of new-type urbanization and the rural digital economy

In developing countries, urbanization is usually defined as a migration from rural to urban and the transformation in demographic, economic and social characteristics [1]. In other words. The main target of urbanization is to concentrate population in cities or large towns, improve land use income and upgrade industrial structure (e.g., increase in industrialization or service industry) [2]. The number and scale of cities are growing unprecedentedly in developing countries [3]. According to the United Nations statistics, the world’s urban population is growing nearly five times at an average rate of 2.6% per year, which is mainly contributed by the developing countries [4]. For instance, in China, the rapid economic growth that has taken place since 1978 has been paralleled by rapid urbanization [5]. According to the National Bureau of Statistics of the PRC, China’s urbanization rate has increased from 17.92% in 1978 to 64.72% in 2021. The high-speed extensive urbanization mode has given rise to a series of issues, such as “urban disease”, “semi-urbanization”, expanding urban-rural divide and environmental degradation [6,7] Many cities are confronting expanding challenges from population growth [8]. Rapid urban growth throughout the developing world has and will continue to pressure environmental, economic, and social systems to provide adequate services for their citizens [9]. In this connection, urbanization is described as a threat to the sustainable development in developing countries, particularly in Latin America and Africa [10]. The challenges brought by urbanization to developing countries has led to a consensus on incorporating the concept of sustainable development into the urbanization process. A unilateral focus of population urbanization cannot meet the requirements for sustainable development of a region [11]. Over the last decade, researchers, policymakers, practitioners, educators and nongovernmental organizations (NGOs) experts have been committed to formulating policies and devising approaches for promoting “sustainable urbanization” [2]. Moreover, there has been a rising trend in this research area [12].

Under such circumstances, *The National New-type Urbanization Plan* (2014–2020) released by the Chinese government on March 16, 2014 initiated new approaches to urbanization in China. This is believed to have started a new era for China’ s urbanization process, which targets people-centered and environmentally friendly pathways. New-type urbanization aims to achieve four major tasks, including expanding domestic demand, enhancing urbanization quality, promoting ecological progress, and coordinating rural-urban relations [13]. This marks that China’s urbanization has entered a new type of multi-target heterogeneous situation [14]. In sharp contrast to traditional urbanization, which centers on the agglomeration of population and economic (as well as social) activities in cities, new-type urbanization emphasizes the balanced geographical distribution of population and economic (as well as social) activities. New-type urbanization stands out from previous policies by emphasizing a “people-centered” and “sustainable” approach [15–19]. The “people-oriented” principle mandates that cities should serve the interests of the people, which stands as the core tenet of the new-type urbanization process [20]. The “sustainable” aspect is manifested in the sustainable and coordinated progress of matters related to the economy, population, society, and the environment [21,22]. Although the formulation of new-type urbanization was initially formulated in China, its goal of attaining coordinated and sustainable development is in accordance with the international concern of sustainable urbanization [20].

The digital economy has increasingly emerged as a crucial engine for economic growth. Its robust development momentum has permeated all economic and social sectors. There is no consensus on the definition of digital economy. This is because our understanding of it has been evolving with its development over time [23]. Initially, the digital economy was defined mainly based on two key elements: ICT (information and communication technology) and e-commerce [24]. As research has advanced, the rapid development of new digital technologies, such as big data, the Internet of Things, cloud computing, artificial intelligence, and blockchain, has enriched the connotation of the digital economy [25–28]. Many institutions and scholars have put forward definitions for the digital economy. In line with *The G20 Digital Economy Development and Cooperation Initiative* adopted at the Hangzhou Summit, it is stated that “The digital economy refers to a series of economic activities which take the utilization of digital knowledge and information as the key factors of production, the modern information network as the important carrier, and the effective application of information and communication technology as the significant driving force for efficiency improvement and economic structure optimization” [29], with various new business models serving as its manifestations [30]. Academics generally concur with this definition, and it is also employed in this study.

According to *The White Paper on China’s Digital Economy Development (2021)* released by the China Academy of Information and Communications Technology [31], the industrial scale of China’s digital economy reached 53.9 trillion RMB in 2023, accounting for 42.8% of the GDP of that year. The integration of the digital economy with various sectors has emerged as a major trend in the new era [32]. In 2022, the Cyberspace Administration of China together with other ten departments issued *the Action Plan for Digital Rural Development (2022–2025)*, clearly stating the efforts that should be made in the development of the rural digital economy. Against this backdrop, the rural digital economy has emerged and is gradually evolving into a new impetus for rural economic development [33,34]. Digital infrastructure has gradually covered most towns and key administrative villages in China, and digital technology has brought substantial transformations in rural areas across various aspects such as agricultural production and circulation, rural e-commerce, rural finance, rural tourism, rural governance, and other rural domains [35–39]. Owing to its multiplier and inclusive effects, the rural digital economy has been playing a pivotal role in galvanizing the rural endogenous driving force and propelling the high quality development and fundamental transformation of traditional agriculture [40].

In comparison to the digital economy, research on the rural digital economy remains relatively scarce. Some scholars try to define the connotation of the rural digital economy. For instance, Mu and Ma considered that the rural digital economy refers to economic activities based on digital infrastructure and using digital technology to promote the agricultural production and economic development in rural areas [22,23,41]. Wu et al. (2022) defined the rural digital economy as a series of economic activities within the policy ambit of the digital economy. Here, digital infrastructure serves as the carrier, digital technology acts as the driving force, and the transformation of rural economic development momentum is regarded as the fundamental approach, thereby enabling the digital transformation of agriculture and the digital-enhanced quality of farmers’ lives [42]. Yang and Li (2023) defined the rural digital economy as an economic form that uses the rural information network as the carrier and digital technology as the impetus. By introducing data elements into the agricultural industry, it promotes the high-quality development of the rural economy through rural industry digitization and rural digital industrialization [43]. Despite the differences in these definitions, the underlying understanding of the rural digital economy is quite similar. That is, the rural digital economy takes digital infrastructure as the carrier, digital technology as the driving force, and rural industry digitization and rural digital industrialization as the crucial manifestations.

### Rural digital economy is a new way to promote new-type urbanization

Scholars have conducted analyses regarding the influencing factors of new-type urbanization from diverse perspectives. They have identified several driving forces behind new-type urbanization, including policy systems, economic development, optimization of industrial structure, scientific and technological innovation, environmental regulation, population flow, and other elements [44–48]. Specifically, policy system and economic development level exert fundamental influences on new-type urbanization [49,50]. The optimization of the industrial structure serves as a significant driving force for urbanization. Moreover, the narrowing of the income gap between urban and rural residents as well as the increase in labor mobility can directly and rapidly boost the urbanization rate [51]. Scientific and technological innovation and environmental regulation are capable of substantially enhancing the quality of urbanization and alleviating the negative impact of urbanization on the environment [52]. Apart from external economic and social factors, in accordance with the core concept of “people-centered” in new-type urbanization, population quality and culture can also have an impact on the level of new-type urbanization [53].

New-type urbanization is in the tide of digital economy development. The green attributes of the digital economy, such as lower marginal cost [54], less resource consumption and environmental pollution are in accordance with the essential demand of new-type urbanization for sustainable development [55]. This makes the digital economy the primary factor and a new driving force influencing future urbanization development [56]. In the existing literature, abundant research has verified the impact of the digital economy on local economic growth [57,58], industrial structure upgrading [59], environmental governance [60–62], and public service supply [63]. In the context of new-type urbanization, Zhang and Zhou (2022) analyzed the impact of digital technologies and policies on China’s in situ urbanization [64]. Hao and Zhao (2025) revealed that digital finance helps promote spatial urbanization [65]. Chen et al. (2022) and Yang et al. (2024) indicated that the digital economy has a positive impact on new-type urbanization by promoting industrial structure upgrading [55,66]. Xu et al. (2021) revealed the internal mechanism of the digital economy on new-type urbanization, namely economic quality, public resources, and the ecological environment [56].

The “Two-Wheel Drive strategy” of rural revitalization and new-type urbanization is a huge proposition for China’s high-quality economic development [67]. Nevertheless, although there are significant differences between the context and focus of these two strategies, there is a mutual-promoting relationship between them. The Marxist theory of urban-rural relationship clarifies the internal logic of the integrated development of urban and rural areas. This theory provides theoretical guidance for the coordinated development of new-type urbanization and rural revitalization [68]. Based on theoretical and empirical analysis, scholars have verified the coupling relationship and long-term synergy between rural revitalization and new-type urbanization [69,70]. The development of new-type urbanization requires various preconditions, and rural development is an important one [71]. Therefore, it is a meaningful topic to explore how to enhance the “driving force” of rural development on new-type urbanization in the context of the rural digital economy.

Although studies have shown that the rural digital economy lagged behind that in cities because of the backward rural digital infrastructure, resulting in the digital divide between urban and rural areas [72,73], the digital economy has played an important role in increasing farmers’ income, expanding employment paths, and promoting urban-rural integration [74]. Some academic studies have delved into the impact of agricultural technology on urbanization. It is believed that agricultural technology facilitates the transfer of the rural surplus labor force, which serves as a necessary condition for urbanization [75]. Jiang (2022) et al. have verified the influence and underlying mechanism of digital agriculture on urbanization [71]. Meanwhile, some scholars have discussed the role that rural e-commerce plays in helping break through the dilemma of urbanization in rural China. It has been verified that the Taobao platform and live e-commerce in China play a significant role in bringing the achievements of urbanization and informatization to rural areas, thereby facilitating the urban-rural integration of infrastructure and public service [76–79]. Ravis and Notkin (2020) put forward that the rural digital economy gathers materials, energy, and labor force, contributing to the rapid expansion of urbanization [80]. Xuan (2022) et al. suggested that the rural digital economy enhances farmers’ knowledge level, expedites the transfer of rural surplus labors to cities, and propels the urbanization of both the population and the economy [81]. Lin (2023) pointed out that the rural digital economy facilitates the deep integration of rural industries and boosts farmers’ income [82]. Yang et al. (2023) verified that there exists a positive influence between new-type urbanization and the rural digital economy, and that new-type urbanization has a greater impact on the rural digital economy [83]. Consequently, the rural digital economy can not only invigorate rural revitalization but also facilitate industrial upgrading and transformation. Moreover, it functions as a driving force to reinforce urban-rural linkages [84,85]. Nevertheless, there is a lack of analyses regarding the overall impact and the underlying mechanism of the rural digital economy on new-type urbanization from a macro perspective.

New-type urbanization endeavors to ensure that all citizens can reap the benefits of urban development. Nevertheless, social inequality might impede the achievement of this objective. The Skill Bias Theory highlights the role of technological change in shaping income inequality [86–88], especially in the context of the digital economy [89,90]. However, academics have not yet agreed on the impact of the digital economy development on income disparities [91]. In developing countries, the most prominent manifestation of social inequality is the urban-rural income gap [85]. This gap serves as the primary impetus for the migration of the rural labor force to urban and town areas. Given the substantial disparity between urban and rural development in China, the growth of the digital economy is prone to exacerbate urban-rural inequality, thereby thwarting the sustainable development of urbanization. Nonetheless, the majority of existing studies analyze the role of social inequality in urbanization from the perspective of the urban-rural income gap, while overlooking the influence of income disparities within rural areas on urbanization.

Another potentially significant constraint within new-type urbanization is rural poverty. Poverty in developing countries typically exhibits the following characteristics: rural-urban disparity, unemployment, poor health, gender discrimination, and ineffective governance [92]. Due to these factors, the rural population is bound to migrate to cities in order to enhance employment opportunities and income levels, as well as to access better social and cultural resources. Nevertheless, during the continuous population shift, social inequalities (between urban and rural areas, between the wealthy and the poor, and between the young and the elderly) will expand [93]. The large scale urbanization and prevalent rural poverty in developing countries, which tend to deteriorate during economic downturn cycles, are increasingly posing a threat to sustainable economic and social development [94–97].

Theoretically speaking, the digital economy, serving as a powerful driving force for economic development, has the potential to boost farmers’ incomes and empower rural areas to address relative poverty. However, there are two contrasting viewpoints regarding the impact of the digital economy on poverty reduction. One perspective holds that the development of the digital economy can lower the Gini coefficient and alleviate poverty [98]. The other view suggests that the digital economy exacerbates poverty. As the information gap widens, the population in poor areas will be at a digital disadvantage, thereby further increasing the gap between the rich and the poor [99].

### Research purpose and possible contributions

This paper aims to examine the impact of the digital economy on China’s new-type urbanization along with its transmission mechanism. The digital economy and new-type urbanization are analyzed within the same framework to address whether the digital economy promotes new-type urbanization in China. If so, this study also examines whether such promotion varies according to geographical region, poverty level, demographic structure, and social equality. Additionally, the research endeavors to identify the mechanisms through which the rural digital economy influences new-type urbanization. To achieve such research purposes, this study quantitatively verifies the impact and mechanism of the rural digital economy on new-type urbanization from multiple perspectives of direct, indirect, and heterogeneous influences. Specifically, the evaluation index systems for the rural digital economy and new-type urbanization are established respectively. Subsequently, based on the panel data of 31 provincial-level administrative regions in China (excluding Hong Kong, Macau, and Taiwan) from 2014 to 2022, the development levels of the rural digital economy and new-type urbanization at the provincial level are measured. A two-way fixed effects model is employed to verify the impact of the rural digital economy on new-type urbanization, as well as its structural and spatial heterogeneity. Finally, a mediation model is utilized to test the mediating roles of rural human capital improvement, agricultural economic growth, rural industrial structure upgrading, and urban-rural market integration, aiming to explore the mechanisms by which the rural digital economy exerts an impact on new-type urbanization.

The possible contributions of this study are outlined below: First, this study places the rural digital economy and new-type urbanization within the same analytical framework and verifies, through empirical analysis, the promoting effect of the rural digital economy on new-type urbanization. In doing so, it explores the paths of new-type urbanization from a new perspective of the rural digital economy. Second, this study breaks new ground by incorporating mediating variables such as rural human capital improvement, agricultural economic growth, rural industrial structure upgrading and urban-rural market integration into the mediation mechanism analysis. By doing so, it is deepening the understanding of the mechanisms through which the rural digital economy promotes new-type urbanization. This enables a more comprehensive assessment of the practice paths of rural development affecting new-type urbanization in the context of the digital economy. Third, the study also conducts a multi-level research to analyse the spatial heterogeneity across provincial-level administrative regions in China by considering variations in institutional frameworks and economic environment. By examining how institutional and economic differences shape the relationship between the rural digital economy and new-type urbanization, the study provides valuable insights into the contextual factors that influence this relationship. This in-depth analysis of inter-provincial differences enhances the applicability and relevance of the findings to policymakers and researchers.

### Theoretical mechanism and hypothesis

**Fig 1:**
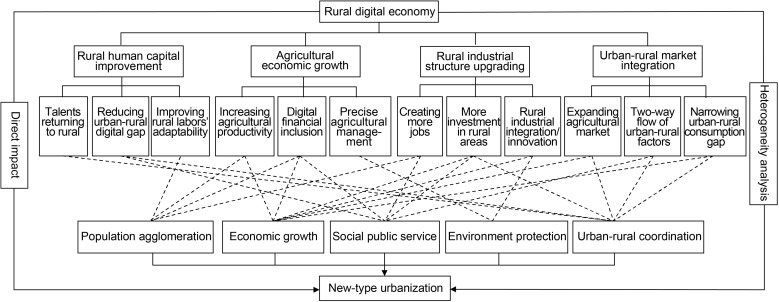
Theoretical mechanism diagram.

By collating relevant research findings and aligning these findings with the research question of this study, four transmission pathways through which the rural digital economy impacts new-type urbanization are proposed, namely rural human capital improvement, agricultural economy growth, rural industrial structure upgrading, and urban-rural markets integration (as illustrated in Fig 1).

Hypothesis 1: The rural digital economy impacts on new-type urbanization through rural human capital improvement

New-type urbanization is people-centered [100]. With the continuous progression of urbanization, the role of human capital will become even more prominent [71]. This mechanism is manifested in the following respects.

Firstly, the rural digital economy is currently at its nascent stage of development and has an enormous demand for high-quality rural talents. Spurred by the rural revitalization strategy, industries related to the rural digital economy have drawn a substantial influx of talents migrating to rural regions, thereby enhancing the level of rural human capital [101]. Secondly, the rural digital economy markedly facilitates the acquisition of rural knowledge, information, and technology by affording more learning and educational opportunities [71]. The incessant renewal and emergence of new digital technologies and novel business models impel farmers to engage in learning and substantially diminish the “digital divide” between urban and rural areas [102]. Consequently, the introduction and cultivation of rural talents augments the level of rural human capital and holds great significance in narrowing the urban-rural gap and fostering coordinated development between urban and rural areas. Furthermore, the rural digital economy bolsters the adaptability of the rural labor force to the job market and expedites the transfer of rural surplus labor to cities [71]. High-quality rural laborers amass and assimilate into cities and towns, circulating among diverse industries, further propelling population urbanization and playing a pivotal role in urban economic and industrial development.

In conclusion, the rural digital economy impacts new-type urbanization via rural human capital improvement, which is achieved by both introducing and cultivating rural talents and enhancing the adaptability of the rural labor force.

Hypothesis 2: The rural digital economy impacts new-type urbanization through agricultural economic growth

The role of the rural digital economy on agricultural economic growth has been proved in existing literature [103–105]. The rural digital economy reshapes rural economic structure [106], optimizes agricultural resources allocation, innovates agricultural development modes, and promotes the transformation from traditional agriculture to modern agriculture [107]. This mechanism is manifested in the following aspects.

First, it improves agricultural productivity. The prevalence of the digital economy allows production factors to flow in a wider-scale space, alleviates the spatial-temporal constraints on economic development, and enhances the quality of economic development through multiple channels, such as cutting transaction costs, preventing resource misallocation, and fostering innovation [108]. The rural digital economy boosts agricultural productivity not only by applying modern technologies to enhance the technical efficiency of traditional factors like labor, land, and capital, thus significantly altering the ratio of agricultural inputs and outputs [109], but also by introducing new production factors such as big data [71]. The increase in agricultural productivity eases the transfer of the rural labor force to other industries and promotes industrial structure upgrading (the expansion of the secondary and tertiary industries), which are essential prerequisites for urbanization [110,111].

Second, it alleviates the financial constraints in the agriculture sector. Digital inclusive finance (DIF) is one of the most effective ways of providing convenient financing support for agricultural development [112]. DIF not only significantly stimulates the supply of agricultural products [113]. By offering more comprehensive financial services to all farmers, it boosts farmers’ enthusiasm for agricultural production [114]. Moreover, DIF promotes high-quality agricultural development by providing new impetus for green agricultural development [115] and the extension of agricultural multifunctionality [113]. Additionally, DIF facilitates the non-farm employment of rural laborers [116], the integration of rural industries [117], rural economic development and the improvement of social security [113], which are important aspects of new-type urbanization.

Furthermore, it helps achieve precise agricultural management. On the one hand, digital agriculture, based on digital technology, promotes agricultural economic growth by reducing production costs. On the other hand, digital agriculture realizes harmless, recycling, and ecological agricultural production through reducing the use of ecological resources, decreasing pesticide pollution, and achieving zero-discharge and full consumption of agricultural wastes. The realization of the agricultural ecological function will be beneficial to society and relieve the constraints of natural resources on economic development [118]. This, in turn, ultimately determines the maximum scale and sustainability of economic growth [119].

In summary, the rural digital economy contributes to agricultural economic growth by enhancing agricultural production efficiency, offering convenient financial support, and realizing precision agriculture. In line with Gunnar Myrdal’s “Cumulative Causation Theory”, the continuous growth of the rural economy will draw in surrounding capital, technology, talents, and other crucial production factors constantly converging towards rural areas. This will significantly boost rural economic vitality, facilitate the integration and balanced development of urban and rural areas, and consequently propel the progress of new-type urbanization.

Hypothesis 3: The rural digital economy impacts new-type urbanization through rural industrial structure upgrading

Driven by digital technology, traditional industries are undergoing a transformation towards Internet-integration and servitization [120]. The digital economy facilitates the formation of a more rational layout of agriculture, industry, and the service sector [121,122]. In particular, its promoting effect on the tertiary industry is more prominent [123].

Under the digital economy, the upgrading of the rural industrial structure encompasses two dimensions. Firstly, there is the digitization of rural industries. The integration of the rural digital economy with various rural industries promotes the digitization, automation, and intelligence of these industries, giving rise to diverse new agricultural production models, such as smart agriculture. Secondly, the industrialization of the rural digital economy is another key aspect. Data elements and digital technologies have spawned a wide range of new business models. Consequently, this facilitates the emergence of some new rural industries that offer digital services to traditional industries [124].

The upgrading of the rural industrial structure leads to the development of rural secondary and tertiary industries. This development generates more job opportunities and promotes the non-farm employment of rural laborers [125]. Beyond the rural population, the upgrading of the rural industrial structure facilitates the flow of production factors from rural areas to cities, thereby promoting urban economic growth [126]. Moreover, with the upgrading of the rural industrial structure, a large number of scaled, organized, and specialized agricultural operation entities emerge. These entities attract more investments in rural areas, thus accelerating rural economic development [127]. Finally, the rural digital economy promotes the integration of agriculture, tourism, culture, and other industries. This integration fosters innovative business forms such as leisure, cultural, and creative agriculture, which meet the spiritual and cultural needs of urban residents [55].

Therefore, the rural digital economy promotes the transformation and upgrading of the rural industrial structure through the digitization of rural industries and the industrialization of the rural digital economy. Subsequently, it has an impact on the urbanization of population, land, economy, space, and ecology.

Hypothesis 4: The rural digital economy impacts new-type urbanization through urban-rural market integration

The digital economy is an important accelerator in the coordinated development of urban and rural areas [128]. It propels urban and rural markets from a state of segmentation towards a unified national-scale market. This mechanism is manifested in the following aspects.

First, the rural digital economy expands the agricultural products market. Specifically, the integration of the urban-rural market is first the connection between agricultural products and urban markets [129]. By adopting some new sales channels such as e-commerce, live streaming, and short-video dissemination [130], the rural digital economy eliminates the congenital disadvantage of the primary industry and remote geographical location [71], and reduces the information asymmetry between urban and rural areas.

Secondly, the rural digital economy promotes the two-way flow of factors between urban and rural areas. In traditional urbanization, surplus rural labor force, natural resources, and other factors are concentrated in urban areas. However, it is rather difficult for urban factors to flow to rural areas. China’ s rural development is faced with the dilemma of lacking production factors and sustainable development capacity, which further widens the urban-rural gap [131]. The rise and rapid development of the rural digital economy have brought about changes in the regional development pattern, resource allocation modes, and subject interests. This drives production factors such as talents, technology, and capital to flow to rural areas, thus promoting the integration of the urban-rural factor market.

Thirdly, the rural digital economy improves rural income and narrows the urban-rural consumption gap. The rural digital economy increases income by expanding the income channels of rural households and reducing production and operation costs [132]. Some studies have revealed a significant reduction in the rural-urban income gap due to the development of the digital economy [133]. Rural e-commerce is a new circulation form of agricultural products outside of wholesale markets, fairs, and supermarkets [134]. It is playing an unprecedented leading role in product publicity, market guidance, and price discovery. By creating a more open and transparent market environment, it alleviates the contradiction between “small farmers” and “big market” to a large extent [71]. In terms of consumption, the increase in rural income stimulates rural consumption upgrading. The transformation from traditional offline consumption to online consumption facilitates the flow of information and commodities between urban and rural areas [135], which narrows the urban-rural consumption gap [136].

Generally speaking, the rural digital economy promotes new-type urbanization through urban-rural market integration by means of expanding the agricultural products market, promoting the two-way flow of urban and rural factors, and narrowing the urban-rural income and consumption gap.

## Methods

### Variables description

#### Explanatory variable.

Although the concepts of the digital economy have been clearly defined, its assessment remains challenging, particularly in the quantitative research on the rural digital economy. The methods for evaluating the level of the digital economy can be classified into two categories based on the evaluation objects. One is direct evaluation, which assesses the level of the digital economy by measuring two core sectors: ICT and e-commerce [24]. However, some scholars argue that direct evaluation fails to cover potential digital economic activities or outcomes [137,138].

Most studies resort to indirect approaches through the construction of indicator systems to gauge the digital economy level across various regions. Considering that the digital economy is endowed with rich connotations and thus calls for a multidimensional assessment, the indicators utilized are intricate and diverse. Nevertheless, the majority of these indicators subsume three dimensions: digital infrastructure, industrial digitization, and digital industrialization [61,139–142].

Among these, digital infrastructure serves as the technical carrier that provides basic support for the digital economy. It is usually evaluated by indicators such as the number of Internet users, broadband access, and the number of mobile base stations. Industrial digitization refers to the application of digital technology in different industries. It is generally estimated by GDP or some indirect indicators, such as the number of R&D or IT employees and the quantity of related equipment or services. Digital industrialization refers to the forerunner industries of the digital economy, encompassing the electronic information manufacturing industry, the software and information technology services industry, the Internet industry, and the telecommunications industry. Several indicators are commonly employed, such as the express delivery volume, the number of official organizational websites, and e-commerce sales volume. The above mentioned three dimensions also offer a direction for the selection of evaluation indicators for the rural digital economy level in this study [143–145].

The existing indicator systems for the digital economy place greater emphasis on high-end digital industries and the in-depth application of digital technology. However, these characteristics are less prominent in rural digital economies and cannot be mechanically replicated. When choosing evaluation indices for the rural digital economy, it is necessary to differentiate between rural and urban areas, as well as between agriculture and industry, in order to establish an evaluation index system that reflects the characteristics of current rural development. *The Evaluation Report of County-Level Digital Agriculture and Rural Developmen*t in 2019, released by the Ministry of Agriculture and Rural Affairs of China, constructed an evaluation index system for digital agricultural and rural development from seven dimensions: development environment, infrastructure support, information consumption, production informatization, operation informatization, service informatization, and governance informatization. This report provides an official and authoritative reference for evaluating the level of the rural digital economy in China.

Referring to the index selection in related literature and the rural digital economy related indices released by authoritative institutions, and taking into account data relevance and availability, this study constructs an evaluation indicator system for the rural digital economy. The system consists of a total of 20 indicators across four dimensions: rural digital infrastructure (*Dige-base*), digital transformation of agriculture (*Dige-agri*), agricultural production service informatization (*Dige-serv*), and digitization of rural life (*Dige-life*) (see Table 1).

**Table 1:** Evaluation index system of the rural digital economy.

First-level indicator		Secondary indicator	Indicator description	Attributes
Rural digital infrastructure (Dige-base)		Rural broadband access households	Number of rural broadband access households (ten thousand)	^+^
		Agricultural meteorological observation station	Number of environmental and agrometeorological observation stations (EA)	^+^
		Rural smart phone penetration rate	Number of mobile phones per 100 rural households at the end of the year (EA)	^+^
		Coverage of radio and television networks	Actual number of rural radio and television users/ total number of rural households (%)	^+^
Rural industrial digitization	Digital transformation of agriculture (*Dige-agri*)	Scale of agricultural digitization	The added value created by the digital economy (RMB: 100 million yuan)	^+^
		Agricultural machinery inputs level	Total power used by all kinds of agricultural machinery (ten thousand kilowatts)	^+^
		New forms of agricultural business	Facility agriculture area (ha)	^+^
		Electrification of agricultural production	Rural electricity consumption (100 million KWH)	^+^
		Labor productivity in the primary industry	Added value of primary industry/ number of employees in primary industry (%)	^+^
		Social digital assets investment	Fixed assets investment in scientific research and technology service industry (RMB: 100 million yuan)	^+^
	Agricultural production service informatization (*Dige-serv*)	Level of agricultural machinery service personnel	Number of personnel in agricultural machinery service organization (person)	^+^
		Level of agricultural machinery service institution	Number of agricultural machinery service organization (EA)	^+^
		Agricultural industry chain extension	The sum of main business income of agricultural and sideline food processing industries (12 industries) (RMB: ten thousand yuan)	^+^
		Agricultural assets investment growth	Growth rate of fixed assets investment in agriculture, forestry, animal husbandry and fishery (RMB: ten thousand yuan)	^+^
		Development level of agricultural production service	Output value of production services in agriculture, forestry, animal husbandry and fishery/ total output value of agriculture, forestry and animal husbandry and fishery(%)	^+^
Rural digital industrialization	Digitization of rural life (Dige-life)	Rural delivery level	The length of the route delivered to rural users (kilometer)	^+^
		Retail level of rural consumer goods	Retail sales of rural consumer goods/ total retail sales of consumer goods (%)	^+^
		Rural digital transaction level	E-commerce sales volume (RMB: 100 million yuan)	^+^
		Development level of digital inclusive finance	Digital financial inclusion index released by The Digital Finance Research Center of Peking University (index)	^+^
		Rural information accessibility level	Total business volume of post and telecommunication (RMB: 100 million yuan)	^+^

First, rural digital infrastructure (*Dige-base*), which serves as the carrier for various rural digital economy activities, represents the fundamental entry threshold for the rural digital economy.

Next, this study characterizes the main impacts of rural industrial digitization from two dimensions, namely, the digital transformation of agriculture (*Dige-agri*) and agricultural production service informatization (*Dige-serv*). The digital transformation of agriculture (*Dige-agri*) mainly reflects the enhancement in agricultural outputs and efficiency brought about by digital technology. Six secondary indicators are selected to reflect the level of production informatization and operation informatization in agriculture. Among these six indicators, the “scale of agricultural digitization” appears to be more complex. In this study, it is calculated by theoretically separating out the contribution of the digital economy from the outputs of the primary industry. Specifically, the added value generated by the digital economy is determined by using the proportion of the intermediate inputs of digital products and services to the total intermediate inputs in the primary industry. The equation is:


$$C=\frac{I_{d}}{I}$$ (1)

$$A_{dav}=C\times A$$ (2)

*C*is the adjustment coefficient of the digital economy in the primary industry; $I_{d}$is the intermediate inputs of digital products and services in the primary industry; *I*is the total intermediate inputs in the primary industry; $A_{dav}$is the added value of the digital economy in the primary industry; *A*is the added value of the primary industry.

Another indicator chosen to reflect rural industrial digitization is the agricultural production service informatization (*Dige-serv*). A well developed agricultural production service system offers more high quality and professional services to agriculture and enables the digitization of agricultural services.

Digital industrialization reflects the capacity of digital infrastructure and industry to support life and production. Indicators that can comprehensively describe rural digital industrialization are extremely scarce. In this study, five secondary indicators are selected from the dimension of digitization of rural life (*Dige-life*) to illustrate the influence of digital-related industries on rural life.

#### Explained variable.

Traditional urbanization adopts a single-indicator measurement method which regards the urbanization rate of the population as the level of urbanization. It emphasizes only the number and scale of cities, while ignoring both environmental costs and development quality. This idea only provides one-sided information in the process of urbanization rather than comprehensively. Since urbanization is a dynamic, multidimensional socio-spatial process, great efforts should be made to achieve a series of targets in the economy, population, society, and environment. Therefore, it is necessary to build a systematic and comprehensive evaluation system to fully reflect the overall level of new-type urbanization.

In terms of sustainable urbanization, researchers have dedicated efforts to developing indicators across environmental, economic, and social dimensions. These indicators aim to guide the sustainable urbanization practice towards the mission of sustainable development [146–149]. Since the concept of new-type urbanization was first put forward, numerous new-type urbanization index systems have been proposed [150–154]. How to measure the level of new-type urbanization has not yet formed a unified consensus; however, reflecting the core features of sustainable, high-quality, and coordinated development is the key to the construction of the new-type urbanization evaluation system.

It is essential that people are at the core of new-type urbanization. Unfolding this idea requires more responsible practices, including creating more jobs, boosting revenue and consumption, and providing adequate social security and services for migrants. Therefore, planning related to economic base, industrial structure, population agglomeration and public service ensures a better life in the city and surrounding areas. This core feature of “people-centered” is manifested in the indicators from the dimensions of population agglomeration (*Peo*), economic growth (*Eco*) and social public service (*Sco*). Another important feature of new-type urbanization is sustainability. Rapid urbanization is inevitably linked to environmental problems and urban-rural gap, which are significant in developing countries. Towards sustainable urbanization, more attention should be given to rural areas and low-carbon economy or ecological economy should be developed [155]. In this study, this feature is manifested in two dimensions: environmental protection (*Env*) and urban-rural coordination (*Coo*).

To sum up, this paper identifies five fundamental dimensions of new-type urbanization, namely population agglomeration (*Peo*), economic growth (*Eco*), social public service (*Soc*), environmental protection (*Env*) and urban-rural coordination (*Coo*), and they jointly characterize the development status of new-type urbanization. Based on this, a systematic evaluation index system is established with a total of 18 secondary indicators selected after considering comprehensiveness, availability, measurability and objectivity, as shown in Table 2.

**Table 2:** Evaluation index system of new-type urbanization.

First-level indicator	Secondary indicator	Indicator description	Attributes
Population agglomeration (Peo)	Urbanization rate	Urban population/ total population (%)	^+^
	Urban population density	Urban population/ urban area (Ten thousand people/ sq^2^)	^+^
	Non-farm employment	Number of employment in secondary and tertiary industries/ total employment number (%)	^+^
	Urban unemployment rate	Registered urban unemployment rate (%)	–
Economic growth (Eco)	Economic development level	Per capita GDP (yuan/ person)	^+^
	Life quality of residents	Per capita disposable income of urban residents (yuan/ person)	^+^
	Industrial development level	The proportion of secondary and tertiary industries in GDP (%)	^+^
	Retail level of consumer goods	Total per capita retail sales of consumer goods (yuan/ person)	^+^
Social public service (Soc)	Investment level in education	Proportion of education expenditure in government expenditure (%)	^+^
	Cultural quality of the population	Years of education per capita (year/ person)	^+^
	Urban public service capacity	Public transport vehicles per 10,000 people (vehicles/ ten thousand persons)	^+^
	Urban infrastructure conditions	Total area of urban roads/ total urban population (square meters/ person)	^+^
Environmental protection (Env)	Environmental pressure	Discharge of industrial wastewater (ten thousand tons)	^+^
	Environmental governance capacity	Harmless treatment rate of household garbage (%)	^+^
	Ecological infrastructure	Park green area per capita (m^2^/person)	^+^
	Farmland protection level	Farmland area (thousand hectares)	^+^
Urban-rural coordination (Coo)	Income gap between urban and rural residents	Urban per capita disposable income/ rural per capita disposable income (%)	–
	Consumption gap between urban and rural residents	Urban per capita expenditure/ rural per capita expenditure (%)	–

Specifically, population aggregation and economic growth in cities are the main targets of traditional urbanization. New-type urbanization does not merely pursue the single target of urban population size or the urbanization rate. Instead, new-type urbanization strongly emphasizes factors that influence the life quality of people, such as jobs, income and consumption. Besides, in new-type urbanization, while maintaining a high GDP level, sustainable economy emphasizes the structure and quality of economic development. Accelerating the growth of secondary and tertiary industries and stimulating domestic demand is considered necessary to improve economic structure and quality. Moreover, urbanization is a complex system that involves a lot of social public service problems. The current growing concerns of more than 200 million migrant workers and their families who are included in the urban population statistics, such as equality of education, availability of medical resources, urban public service capacity and infrastructure should be included in new-type urbanization. Furthermore, environmental protection has always been difficult due to the priority given to the economic growth target. New-type urbanization gives priority to the environment, focuses on green development, and adopts some frequently-used solutions to achieve this goal, such as reducing pollution discharge, increasing the urban waste treatment rate, and protecting urban greenery and farmland. Finally, the urban-rural relationship has emerged as a pivotal element of the cityscape [156]. In recent years, with the increasing incidence of urban-rural conflicts, urban-rural integration has been put forward as a crucial link within the framework of new-type urbanization. To bridge the development gap between urban and rural areas, addressing the disparities in income and consumption levels between urban and rural regions has been identified as a top priority.

#### Control variables.

Control variables are specified to ensure the accuracy and reliability of the study results. This helps us ascertain the relationship between the rural digital economy and new-type urbanization during the empirical process as a causal relationship rather than one influenced by other factors.

Degree of openness (*Ope*). The expansion of international trade not only fosters business development but also generates employment, thereby creating more market opportunities [157]. Openness can introduce new technologies and management experiences. These new elements tend to concentrate in higher-yield industries, such as the secondary and tertiary industries. This situation may result in lower wages for rural residents and workers in low-end industries, potentially increasing income inequality [158]. It is measured by the ratio of the regional total volume of imports and exports to regional GDP.

Investment in fixed assets (*Inv*). Fixed-asset investment encompasses aspects such as infrastructure construction, industrial development, and investment in social undertakings, all of which are indispensable components of new-type urbanization. This indicator is measured as the ratio of total fixed-asset investment to regional GDP.

Scientific and technological progress (*Tec*). Innovation is the primary driving force for high-quality development, and the level of scientific and technological progress represents the capacity of new-type urbanization in the region. It is measured as the ratio of R&D expenditure to regional GDP.

Transportation infrastructure (*Tra*). Transportation infrastructure facilitates connections between towns, as well as between towns and villages, which contributes to urban-rural integration and new-type urbanization. Specifically, this index is measured by the ratio of highway mileage to the population size at the end of the year.

Industrial structure upgrading (*Ind*). Urbanization is the inevitable outcome of industrialization [159]. The spatial agglomeration of industrial production leads to the formulation of cities through guiding the agglomeration of the non-agricultural population. Service industry is an important engine for the development and transformation of modern cities. This indicator is measured by the ratio of the added value of the tertiary industry to the added value of the secondary industry.

Rural poverty (*Pov*). There is a close relationship between rural poverty and population mobility. Population mobility contributes positively to poverty alleviation, such as income-increase, employment opportunity expansion, and economic development promotion. However, population mobility may give rise to brain drain in poor areas, thereby affecting local economic development and social stability. Furthermore, the widespread rural poverty in developing countries poses a significant impediment to achieving the new-type urbanization target. This indicator is measured by the rural poverty rate.

Population structure (*Pop*). By reducing population mobility and labor supply, the aging of the population hinders human capital accumulation and industrial structure upgrading [160–163], and then significantly inhibits new-type urbanization [164,165]. According to the endogenous growth theory, the aging population affects regional innovation by significantly crowding out R&D input [166]. In addition, the aging of the population inevitably impacts the scale and structure of consumption [167]. Therefore, this study uses the aging population as a control variable, with the expected result that the aging of the population may hinder new-type urbanization. This indicator is measured by the elderly dependency ratio.

Social inequality (*Equ*). In addition to inter-sectoral inequality and urban-rural inequality [168,169], rural inequality, mainly the rural income disparity, may obstruct the process of new-type urbanization. The widening rural income gap has an obvious negative effect on the low-education and low-income class and families in less developed areas. This may reduce farmers’ human-capital investment, thus inhibiting innovation and entrepreneurship in rural areas [170]. Referring to the calculation method of Gini coefficient by Tian [171], this study calculated the Gini coefficient of rural income to indicate the rural inequality in a region.


$$G=1\text{-}\frac{1}{PW}\sum_{i=1}^{\text{n}}(W_{i-1}+W_{i})\times P_{i}$$ (3)

In Eq (3), the Gini coefficient *G* represents the degree of inequality, with values ranging from 0 to 1. A value of 0 indicates perfect equality, while a value of 1 indicates complete inequality. *n* is the number of groups into which the population is divided when sorted from lowest to highest income. *i* is the index variable for summation, ranging from 1 to *n* and representing the sequence number of the group being calculated. $W_{\text{i}-1}$is the cumulative proportion of the population in the *i-*1_th_ group. $W_{\text{i}}$is the cumulative proportion of the population in the *i*_th_ group. When calculating the Gini coefficient for income distribution, $W_{\text{i}}$represents the proportion of the population in the group with the lowest income to the total population. $P_{\text{i}}$is the proportion of the income of the *i*_th_ group to the total income. *PW* is usually a standardization factor used to ensure that the Gini coefficient ranges from 0 to 1. In common calculations, *PW*=2.

#### Mediating variables.

Based on the theoretical mechanism analysis, this study identified four mediating variables with the aim of clarifying the influencing mechanism of the rural digital economy on new-type urbanization.

Rural human capital improvement (*HC*). This variable is measured by the average nominal rural human capital stock from *The China Human Capital Report 2023* issued by the Center for Human Capital and Labor Market Research [172]. It measures human capital by integrating traditional measures based on education level and the Jorgenson-Fraumeni (J-F) income calculation method based on the internationally universal lifetime income, which can offer more up-to-date and accurate human capital estimations for rural areas.

Agricultural economic growth (*EG*). This variable is gauged by the total output value of agriculture, forestry, animal husbandry, and fisheries. Such a measure comprehensively reflects the overall economic performance and growth level of the agricultural industry.

Rural industrial structure upgrading (*IS*). This variable is gauged by the ratio of the number of rural non-agricultural workers to the total number of rural employees. It serves as an important indicator to reflect the degree of diversification and upgrading of the rural industrial structure.

Urban-rural market integration (*Int*). Nine indicators were selected from both input and output perspectives in this study to comprehensively evaluate the level of urban-rural market integration (see Table 4), and the entropy weight method was used to determine the weight of each indicator. The calculation formula for the index of urban-rural market integration is.

**Table 4:** Indicators of urban-rural market integration.

First-level indicator	Secondary indicator	Indicator description
Integration of input	Integration of income	Urban residents’ disposable income/ rural residents’ disposable income (%)
	Integration of infrastructure	Urban per capita road mileage/ rural per capita road mileage (%)
	Integration of information foundation	Internet penetration rate in urban areas/ internet penetration rate in rural areas (%)
	Integration of financial input	Scale of urban consumer finance/ scale of rural consumer finance (scale of consumer finance is represented by total credit of credit card at the end of the term. Data is from the official website of People’s Bank of China) (%)
	Integration of human capital	Number of retail employees per 10,000 people in urban areas/ number of retail employees per 10,000 people in rural areas (%)
Integration of output	Integration of consumption scale	Urban per capita consumption/ rural per capita consumption (%)
	Integration of consumer price	Urban CPI/ rural CPI (%)
	Integration of consumption structure	Engel coefficient of urban households/ Engel coefficient of rural households (%)
	Integration of consumer tendency	Marginal consumption rate of urban households/ marginal consumption rate of rural households (Marginal consumption rate = increased consumption/ increased income) (%)


$$cond_{it}=\xi_{i}\sum_{i=1}^{m}q_{it}$$ (4)

In Eq (4), ${C\text{ond}}_{it}$is the index of urban-rural market integration of each province, $\xi_{i}$is the weight of each indicator, mis the number of indicators (mis nine in this study), and ${\text{q}}_{it}$is the actual value of each indicator (Table 3).

**Table 3:** Model variables.

Variable type	Variable name	Symbol	Indicator description
Explained variable	New-type urbanization	NTU	Calculated by CRITIC weight method (index)
Explanatory variable	Rural digital economy	Dige	Calculated by Entropy TOPSIS method (index)
Control variables	Degree of openness	Ope	Regional total amounts of imports and exports/ regional GDP (%)
	Investment in fixed assets	Inv	Total investment in fixed assets/ regional GDP (%)
	Scientific and technological progress	Tec	R&D expenditure/ regional GDP (%)
	Transportation infrastructure	Tra	Highway mileage/ population size at the end of the year (%)
	Industrial structure upgrading	Ind	The added value of the tertiary industry/ the added value of the secondary industry.
	Population structure	Pop	Elderly dependency ratio=number of population over 65 years old/ number of population of working age (%)
	Rural poverty	Pov	Rural poverty rate=number of rural poor people/ total number of rural registered population
	Social inequality	Equ	The Gini coefficient of rural income
Mediating variables	Rural human capital improvement	HC	Average rural nominal human capital stock (index)
	Agricultural economic growth	EG	The total output value of agriculture, forestry, animal husbandry and fishery (RMB: 100 million yuan)
	Rural industrial structure upgrading	IS	The number of rural non-agricultural workers/ the number of rural employed persons (%)
	Urban-rural market integration	Int	Index of urban-rural market integration

## Data source

This study collected the panel data of the 31 provincial-level administrative regions in China (excluding Hong Kong, Macao, and Taiwan) from 2014 to 2022. The index data of the rural digital economy are sourced from various materials, including the China Statistical Yearbook, China Agricultural Yearbook, China Industrial Statistical Yearbook, China Third-Industry Statistical Yearbook, China Rural Management Statistical Annual Report, China Population and Employment Statistical Yearbook, National Greenhouse Data System, as well as the statistical yearbooks of the 31 provincial-level administrative regions. For the index data of new-type urbanization, they are obtained from the China Statistical Yearbook, China Rural Statistical Yearbook, Statistical Yearbook of China Urban and Rural Construction, China Human Capital Report (2023), along with the statistical yearbooks and bulletins of the 31 provincial-level administrative regions. The index data of the control and mediating variables are retrieved from the China Statistical Yearbook, China Agricultural Yearbook, China Trade and Foreign Economic Statistical Yearbook, China Human Capital Report (2023), China Household Survey Yearbook, the official website of the People’ s Bank of China, and the statistical yearbooks of the 31 provincial-level administrative regions. The Linear Interpolation Method is used to handle the problem of missing data in some provinces or years.

### Data analysis methods

#### CRITIC.

In the present study, the CRITIC (Criteria Importance Through Inter criteria Correlation) method is employed to assess the development level of new-type urbanization. The CRITIC method is a comprehensive approach that determines the objective weights of indicators by taking into account both the contrast intensity among indicators and the conflict between them. By eliminating the influence of highly correlated indicators and reducing the information overlap between indicators, it ensures the credibility of the evaluation results [173].

The contrast intensity, which is expressed in the form of standard deviation, represents the difference in values within each individual indicator. Mathematically, it can be described as follows:


$$S_{\text{j}}=\sqrt{\frac{\sum_{i=1}^{\text{n}}�{\text{x}}_{ij}-{\bar{x}}_{j}{)}^{2}}{n-1}}$$ (5)

Next, we calculate the quantitative indicator that reflects the conflict between the *j*_th_ indicator and other indicators. Here, ${\text{r}}_{\text{ij}}$is the correlation coefficient between the *i*_th_ indicator and the *j*_th_ indicator.


$$R_{\text{j}}=\sum_{i=1}^{p}�1\text{-}{\text{r}}_{ij})$$ (6)

The amount of information contained in the *j*_th_ indicator is calculated by combining the contrast intensity$S_{\text{j}}$and the conflict indicator$R_{\text{j}}$. Subsequently, the objective weight$W_{\text{j}}$of the *j*_th_ indicator is obtained through the following equation:


$$C_{\text{j}}=S_{j}\sum_{i=1}^{p}(1-r_{ij})=S_{j}\times R_{j}$$ (7)

$$W_{\text{j}}=\frac{C_{j}}{\sum_{i=1}^{n}C_{j}}$$ (8)

Finally, based on the weight $W_{\text{j}}$and the value ${\text{x}}_{ij}^{'}$of the _th_ indicator after dimensionless treatment, the evaluation score $P_{\text{ij}}$of the *j*_th_ indicator is calculated as follows:


$$F_{\text{j}}=\sum_{i=1}^{\text{p}}W_{j}\times x_{ij}^{'}$$ (9)

#### Entropy TOPSIS.

Entropy TOPSIS (Technique for Order Preference by Similarity to Ideal Solution) is employed to measure the development level of the rural digital economy. The concept behind entropy TOPSIS is to utilize the entropy weight method to determine the weight of each indicator, thereby avoiding subjectivity. Subsequently, the approximation to ideal solution technique is applied to establish the ranking of the evaluation object [174].

Step 1: Undimensionization to obtain the normalized matrix. The equation for obtaining the normalized matrix$P_{\text{ij}}$ is:


$$P_{\text{ij}}=\frac{{\text{x}}_{ij}^{'}}{\sum_{i=1}^{m}x_{ij}^{'}}$$ (10)

Step 2: Construction of the weighted decision matrix. After obtaining the normalized matrix, we combine the index weights$w_{j}$to get the weighted decision matrix$R_{\text{ij}}$. The equation is:


$$R_{\text{ij}}=x_{ij}^{'}\times w_{j}$$ (11)

Step 3: Determination of positive and negative ideal solutions for each index. The positive ideal solution$H_{\text{j}}^{+}$and the negative ideal solution$H_{\text{j}}^{-}$are calculated as follows:


$$H_{\text{j}}^{+}=\left\{\max\left\{R_{ij}\right\}\right\}$$ (12)

$$H_{\text{j}}^{-}=\left\{m\text{in}\left\{R_{ij}\right\}\right\}$$ (13)

Step 4: Calculation of euclidean distances. The Euclidean distance$D_{\text{i}}^{+}$from the *i*_th_ evaluation item to the positive ideal solution and $D_{\text{i}}^{-}$to the negative ideal solution are given by:


$$D_{\text{i}}^{+}=\sqrt{\sum_{j=1}^{n}{\left(R_{ij}-H_{j}^{+}\right)}^{2}}$$ (14)

$$D_{\text{i}}^{-}=\sqrt{\sum_{j=1}^{n}{\left(R_{ij}-H_{j}^{-}\right)}^{2}}$$ (15)

Step 5: Calculation of relative closeness and ranking. First, calculate the relative closeness$C_{i}$of each evaluation item using the Eq (16). Then, order the evaluation objects sequentially based on the values of$C_{i}$.


$$C_{i}=\frac{D_{i}^{-}}{D_{i}^{+}+D_{i}^{-}}$$ (16)

#### Global-local spatial auto-correlation analysis.

Spatial auto-correlation analysis is a spatial statistical method that can reveal the regional structural morphology of spatial variables. Global Moran’s I is an overall measurement of the spatial correlation across the entire study area. As a complement to global spatial auto-correlation analysis, local Moran’s I has the ability to disclose the clustering or dispersion of data at diverse spatial locations.

Global Moran’ s I:


$$I=\frac{\text{n}}{S_{0}}\times \frac{\sum_{i=1}^{n}\sum_{j=1}^{n}W_{ij}(y_{i}-\bar{y})(y_{j}-\bar{y})}{\sum_{i=1}^{n}{\left(y_{i}-\bar{y}\right)}^{2}}$$ (17)

Where$S_{0}=\sum_{i=1}^{\text{n}}\sum_{j=1}^{n}W_{ij}$, *n* is the total number of spatial units, ${\text{y}}_{i}$and${\text{y}}_{\text{j}}$represent the attribute values of the spatial unit *i* and *j*, respectively, $\bar{y}$is the mean value of the attribute values of all spatial units, ${\text{W}}_{\text{ij}}$is the spatial weight value.


$$\text{Local Moran’s I:}{\;I}_{i}=\frac{Z_{i}}{S^{2}}\sum_{j\neq i}^{n}w_{ij}Z_{j}$$ (18)

Where${\text{I}}_{i}$is the local Moran index in region *i*,$Z_{i}={\text{y}}_{\text{i}}-\bar{y}$,$Z_{i}={\text{y}}_{j}-\bar{y}$,$S^{2}=\frac{1}{\text{n}}\sum (y_{i}-\bar{y}{)}^{2}$.

Geary’s C is another statistical metric for spatial auto-correlation. It is employed to evaluate the extent of spatial clustering or dispersion of specific geographical data.

Geary’s C:


$$G=\frac{�\text{n}-1)\sum_{i}\sum_{j}w_{ij}{\left({\text{x}}_{i}-x_{j}\right)}^{2}}{2\sum_{i}\sum_{i}w_{ij}{\left({\text{x}}_{\text{i}}-\bar{x}\right)}^{2}}$$ (19)

Where, *G* is Geary’s C index, *n* is the number of samples, is the geographical adjacency weight, is the value of sample *i*, is the value of the sample *j*, and $\bar{\text{x}}$is the average of all samples. The value range of Geary’s C index lies between 0 and 2. When the value of *G* approximates 1, it indicates that the data follow a random distribution. When *G* is close to 0, it implies that the data display a positively correlated spatial clustering. When *G* approaches 2, it suggests that the data exhibit a negatively correlated spatial dispersion.

#### Two-way Fixed Effect Model.

To verify the influence of the rural digital economy on new-type urbanization at the overall level, this study employs the two-way fixed effect model for benchmark regression. Based on the panel data of the 31 provincial-level administrative regions from 2014 to 2022, the following model is utilized:


$${NTU}_{\text{it}}=\alpha_{0}+\beta_{0}{Dige}_{it}+\varphi_{0}X_{it}+\gamma_{i}+\mu_{i}+\varepsilon_{it}$$ (20)

Where *i* is an individual province, *t* is the year, ${NTU}_{it}$is the explained variable, which can be a measure of the development level of new type urbanization in the *i*_th_ province in year *t*. ${D\text{ige}}_{it}$is the explanatory variable, referring to the indicator related to the rural digital economy in the *i*_th_ province in year*t.* $X_{it}$is control variable in the *i*_th_ province in year *t*. $\gamma_{i}$is the time effect, $\mu_{i}$is the individual fixed effect, $\varepsilon_{it}$is the random error.

To address the potential endogeneity issue in the benchmark regression results, appropriate instrumental variables are selected, and the Two Stage Least Squares (2SLS) method is applied for the endogeneity test. Additionally, a robustness test is conducted to ensure the accuracy and reliability of the conclusions drawn from the regression model.

#### Two-stage least squares model.

The sources of endogeneity primarily include reverse causality and omitted variables. This paper attempts to select appropriate instrumental variables and uses Two-Stage Least Squares (2SLS) to estimate the possible endogeneity of the benchmark regression results. Referring to the two-dimensional instrumental variables selected by some scholars [175–177], this study constructs the core instrumental variable based on the product of the number of scientific and technological papers published by universities and topographic relief degree. Additionally, alternative instrumental variable based on the product of the number of websites owned by 100 enterprises and topographic relief degree is also constructed to further validate the robustness of the endogenous test results.

The topographic relief degree affects the infrastructure construction cost and information dissemination efficiency in the region, thus promoting or restricting the development of rural digital economy. Meanwhile, the topographic relief degree is a relatively stable and independent geographical variable, which will not be affected by human factors or other random errors in the process of new type urbanization. The number of scientific and technological papers published by universities can, to some extent, reflect the knowledge innovation capabilities and scientific and technological research and development levels of a region. Knowledge and technological innovation are key factors driving the development of the digital economy. As the important entity for knowledge innovation, universities’ paper-publishing can demonstrate the research achievements and innovation vitality of the region in related fields such as digital technology and information technology, and thus have an inherent connection with the development of the rural digital economy. Moreover, the publication of papers by universities is mainly influenced by internal factors such as the universities’ own scientific research capabilities, and there is no direct causal relationship between it and the error terms that may arise in the process of new urbanization. Using the product of the two as an instrumental variable enriches and rationalizes the relationship between the instrumental variable and the explanatory variable. Meanwhile, since both of them are exogenous in nature, their product also meet the requirement of “strict exogeneity” for instrumental variable, helping us to more accurately identify the causal relationship between the core variable and the explained variable. The alternative instrumental variable—the product of the number of websites owned by 100 enterprises and topographic relief degree also conforms to the above characteristics.

#### Robustness testing methods.

To ensure our results remain consistent across different model specifications or sample selections, thereby verifying result stability and enhancing the persuasiveness of the study, this paper conducts robustness tests from the following perspectives. (1) Replacing calculation method. Previously, the level of the rural digital economy was measured using the entropy TOPSIS method. To verify the robustness of the benchmark regression results, this paper employs the principal component analysis method (PCA) to re-measure the rural digital economy level index and then re-conducts the regression analysis. (2) Excluding special samples. Given that some samples might exert an asymmetric influence on the overall regression results, this paper eliminates the top four provinces in terms of the development level of the rural digital economy and then re-conducts the regression analysis. (3) Winsorization. To control the potential interference from data outliers, the data related to the rural digital economy and new-type urbanization are subjected to bilateral Winsorization at the 1% and 99% levels. (4) Altering sample range. Using 2015 as the cut-off point, the sample data from 2015 to 2021 are retained for re-estimation. In 2015, *The No.1 Central Document* first proposed “rural e-commerce”, marking the beginning of the exploratory stage of the rural digital economy. Thus, the development of the rural digital economy after 2015 is more representative.

#### Mediation effect model.

Based on the above theoretical mechanism analysis, the rural digital economy may exert an impact on new-type urbanization via rural human capital improvement (*HC*), agricultural economic growth (*EG*), rural industrial structure upgrading (*IS*), and urban-rural market integration (*Int*). This study adopts the method proposed by Wen et al. [178] to construct the following mediation effect models for verification. To ensure the reliability of the regression results, both the Sobel test and the Bootstrap test are conducted.


$${NTU}_{\text{it}}=\alpha_{1}+\beta_{1}{Dige}_{it}+\varphi_{1}X_{it}+\gamma_{i}+\mu_{i}+\varepsilon_{it}$$ (21)

$$M_{\text{it}}=\alpha_{2}+\beta_{2}{Dige}_{it}+\varphi_{2}X_{it}+\gamma_{i}+\mu_{i}+\varepsilon_{2it}$$ (22)

$${NTU}_{\text{it}}=\alpha_{3}+\beta_{3}{Dige}_{it}+\eta M_{\text{it}}+\varphi_{3}X_{it}+\gamma_{i}+\mu_{i}+\varepsilon_{2it}$$ (23)

A step-by-step regression method is employed to demonstrate the mediating effect. In this context, $\beta_{1}$represents the total effect of the rural digital economy (${D\text{ige}}_{it}$) on new-type urbanization (${NTU}_{it}$), and $\beta_{3}$is the direct effect of the rural digital economy on new-type urbanization when the mediating variableMitis also included in the model. The mediating effect of the rural digital economy on new-type urbanization through the mediating variable Mitis calculated as$\beta_{2}\times \eta$. The proportion of the mediating effect in the total effect is$\frac{\beta_{2}\times \eta }{\beta_{1}}$.

## Results

Based on the above model construction and acquired data, this study carried out econometric analysis by using Stata 18, and obtained the following empirical research results.

### System measurement results

The development levels of the rural digital economy and new-type urbanization in the 31 provincial-level administrative regions of China were measured by employing the entropy TOPSIS Method and the CRITIC weight method (see Table 5 and 6). The data indicates that the overall development level of the rural digital economy and new-type urbanization in China has been steadily increasing from 2014 to 2022. Moreover, to further clarify the regional differences, according to the China National Bureau of Statistics, the 31 provincial-level administrative regions were divided into four geographical regions, namely the eastern, central, western, and northeastern regions. Specifically, the eastern region includes Beijing, Tianjin, Hebei, Shanghai, Jiangsu, Zhejiang, Fujian, Shandong, Guangdong, and Hainan. The central region includes Shanxi, Anhui, Jiangxi, Henan, Hubei, and Hunan. The western region includes Inner Mongolia, Guangxi, Chongqing, Sichuan, Guizhou, Yunnan, Shaanxi, Gansu, Qinghai, Ningxia, Xinjiang, and Tibet. The northeast region includes Heilongjiang, Jilin, and Liaoning.

**Table 5:** The development level of the rural digital economy in the 31 provincial-level administrative regions of China from 2014 to 2022.

Province	2014	2015	2016	2017	2018	2019	2020	2021	2022
Beijing	0.1189	0.1098	0.1225	0.1359	0.1739	0.1778	0.2080	0.2287	0.2606
Tianjin	0.1016	0.1095	0.1062	0.1337	0.1683	0.1424	0.1463	0.1529	0.1704
Hebei	0.2317	0.2392	0.2411	0.2507	0.2619	0.2829	0.3045	0.3328	0.3453
Shanghai	0.1514	0.1369	0.1902	0.1969	0.1946	0.2062	0.2225	0.2356	0.2674
Jiangsu	0.4064	0.4600	0.4832	0.4922	0.5159	0.5391	0.5634	0.5929	0.6227
Zhejiang	0.1955	0.1925	0.2020	0.2032	0.2159	0.2548	0.2943	0.3169	0.3149
Fujian	0.1269	0.1202	0.1280	0.1378	0.1514	0.1739	0.2022	0.2007	0.2163
Shandong	0.3869	0.3945	0.4345	0.4450	0.4531	0.4430	0.4977	0.5190	0.5377
Guangdong	0.2339	0.2486	0.2606	0.2760	0.3174	0.3612	0.4032	0.4259	0.4677
Hainan	0.0580	0.0644	0.0729	0.0779	0.0868	0.1018	0.1175	0.1321	0.1492
**East**	**0.2011**	**0.2076**	**0.2241**	**0.2349**	**0.2539**	**0.2683**	**0.2959**	**0.3138**	**0.3352**
Shanxi	0.1078	0.1118	0.1141	0.1094	0.1091	0.1158	0.1236	0.1339	0.1384
Anhui	0.1450	0.1569	0.1711	0.1894	0.1946	0.2257	0.2404	0.2638	0.2753
Jiangxi	0.1341	0.1365	0.1434	0.1588	0.1663	0.1825	0.1919	0.2023	0.2028
Hennan	0.2327	0.2432	0.2582	0.2703	0.2817	0.3150	0.3334	0.3594	0.3555
Hubei	0.1590	0.1796	0.1906	0.2005	0.2097	0.2347	0.2533	0.2604	0.2634
Hunan	0.1759	0.1875	0.2049	0.2260	0.2511	0.3107	0.3488	0.3751	0.3915
**Central**	**0.1591**	**0.1692**	**0.1804**	**0.1924**	**0.2021**	**0.2307**	**0.2486**	**0.2658**	**0.2712**
Heilongjiang	0.2340	0.2350	0.2544	0.2588	0.2532	0.2808	0.2959	0.3127	0.3318
Jilin	0.1019	0.1114	0.1236	0.1330	0.1371	0.1493	0.1559	0.1688	0.1863
Liaoning	0.2918	0.2748	0.2725	0.2277	0.2016	0.1810	0.1995	0.2099	0.2227
**Northeast**	**0.2092**	**0.2071**	**0.2168**	**0.2065**	**0.1973**	**0.2037**	**0.2171**	**0.2304**	**0.2469**
Inner Mongolia	0.1120	0.1199	0.1255	0.1284	0.1358	0.1482	0.1588	0.1711	0.1793
Guangxi	0.1032	0.1016	0.1173	0.1214	0.1277	0.1590	0.1806	0.1983	0.2168
Chongqing	0.0999	0.1026	0.1041	0.1022	0.1078	0.1187	0.1310	0.1438	0.1503
Sichuan	0.2148	0.2167	0.2338	0.2413	0.2521	0.2928	0.3080	0.3351	0.3258
Guizhou	0.0666	0.0728	0.0748	0.0813	0.0918	0.1313	0.1408	0.1680	0.1690
Yunnan	0.0911	0.0964	0.1110	0.1136	0.1271	0.1441	0.1564	0.1880	0.2169
Tibet	0.0560	0.0599	0.0618	0.0631	0.0649	0.0678	0.0689	0.0706	0.0754
Shaanxi	0.1050	0.1269	0.1225	0.1326	0.1401	0.1644	0.1684	0.1847	0.1911
Gansu	0.1180	0.1221	0.1309	0.1225	0.1344	0.1338	0.1363	0.1423	0.1445
Qinghai	0.0368	0.0369	0.0431	0.0455	0.0471	0.0545	0.0591	0.0647	0.0671
Ningxia	0.0480	0.0510	0.0556	0.0573	0.0593	0.0590	0.0637	0.0681	0.1529
Xinjiang	0.0880	0.0979	0.1084	0.1161	0.1218	0.1291	0.1406	0.1672	0.1920
**West**	**0.0949**	**0.1004**	**0.1074**	**0.1104**	**0.1175**	**0.1335**	**0.1427**	0.1585	**0.1734**
**National**	**0.1527**	**0.1586**	**0.1698**	**0.1758**	**0.1856**	**0.2026**	**0.2198**	**0.2363**	**0.2516**

Note: First, the evaluation score of *Dige* in each province in each year was calculated using Entropy TOPSIS, then, the average score of each geographical region in each year was calculated based on them.

**Table 6:** The development level of new-type urbanization in the 31 provincial-level administrative regions of China from 2014 to 2022.

Province	2014	2015	2016	2017	2018	2019	2020	2021	2022
Beijing	0.6113	0.6139	0.5986	0.6110	0.6308	0.6245	0.6277	0.6383	0.6320
Tianjin	0.5827	0.6223	0.5853	0.5868	0.6071	0.6013	0.5944	0.6016	0.6161
Hebei	0.4512	0.4534	0.4744	0.4941	0.5204	0.5391	0.5446	0.5471	0.5699
Shanghai	0.5189	0.5197	0.5209	0.5336	0.5520	0.5607	0.5864	0.5977	0.6320
Jiangsu	0.5130	0.5222	0.5383	0.5654	0.5909	0.5918	0.6064	0.6184	0.6473
Zhejiang	0.5117	0.5400	0.5492	0.5637	0.5845	0.5939	0.6049	0.6153	0.6265
Fujian	0.4413	0.4799	0.4922	0.5045	0.5340	0.5524	0.5702	0.5766	0.6143
Shandong	0.4847	0.4993	0.4890	0.5298	0.5459	0.5457	0.5510	0.5636	0.5844
Guangdong	0.4842	0.5023	0.5002	0.5312	0.5468	0.5695	0.5865	0.5954	0.6126
Hainan	0.4101	0.4269	0.4392	0.4379	0.4569	0.4569	0.4683	0.4745	0.5121
**East**	**0.5009**	**0.5180**	**0.5187**	**0.5358**	**0.5569**	**0.5636**	**0.5740**	**0.5829**	**0.6047**
Shanxi	0.4612	0.4634	0.4837	0.4956	0.5031	0.5157	0.5227	0.5378	0.5644
Anhui	0.4150	0.4311	0.4563	0.4531	0.4466	0.4540	0.4470	0.4655	0.4868
Jiangxi	0.4042	0.3990	0.4222	0.4175	0.4397	0.4414	0.4390	0.4573	0.4844
Hennan	**0.4268**	**0.4312**	**0.4541**	**0.4554**	**0.4631**	**0.4704**	**0.4696**	**0.4869**	**0.5119**
Hubei	0.4340	0.4516	0.4684	0.4831	0.4847	0.4995	0.5211	0.5308	0.5665
Hunan	0.4138	0.4551	0.4697	0.4888	0.5116	0.5341	0.5527	0.5682	0.5857
Central	0.4760	0.4898	0.4959	0.5038	0.5414	0.5541	0.5609	0.5751	0.5996
Heilongjiang	0.4436	0.4737	0.4798	0.5106	0.5398	0.5518	0.5607	0.5747	0.5753
Jilin	0.3999	0.4442	0.4577	0.4918	0.5062	0.5129	0.5268	0.5204	0.5551
Liaoning	0.3832	0.4141	0.4229	0.4473	0.4690	0.4849	0.5185	0.5492	0.5804
**Northeast**	**0.4251**	**0.4548**	**0.4657**	**0.4876**	**0.5088**	**0.5229**	**0.5401**	**0.5531**	**0.5771**
Inner Mongolia	0.3994	0.4398	0.4633	0.4858	0.4959	0.4948	0.5312	0.5300	0.5604
Guangxi	0.3287	0.3787	0.4060	0.4267	0.4520	0.4604	0.4696	0.4891	0.5230
Chongqing	0.3976	0.4302	0.4396	0.4541	0.4770	0.4919	0.5086	0.4740	0.5291
Sichuan	0.3897	0.4067	0.4045	0.4348	0.4547	0.4733	0.4942	0.4979	0.5056
Guizhou	0.3286	0.3402	0.3612	0.3907	0.4064	0.4248	0.4382	0.4697	0.4683
Yunnan	0.2947	0.3505	0.3565	0.3897	0.4097	0.4284	0.4375	0.4371	0.4526
Tibet	0.2733	0.3065	0.3566	0.3231	0.3249	0.3287	0.3572	0.4019	0.4281
Shaanxi	0.4698	0.4912	0.4708	0.4871	0.4944	0.4998	0.5261	0.5419	0.5556
Gansu	0.3386	0.3736	0.3918	0.4130	0.4415	0.4260	0.4391	0.4520	0.4705
Qinghai	0.3262	0.3473	0.3515	0.3742	0.4006	0.4166	0.4511	0.4663	0.4888
Ningxia	0.3638	0.4072	0.4139	0.4299	0.4495	0.4549	0.5164	0.5386	0.5443
Xinjiang	0.3808	0.4070	0.4208	0.4328	0.4527	0.4808	0.5384	0.5541	0.5701
**West**	**0.3576**	**0.3899**	**0.4030**	**0.4202**	**0.4383**	**0.4484**	**0.4756**	**0.4877**	**0.5080**
**National**	**0.4237**	**0.4480**	**0.4580**	**0.4746**	**0.4930**	**0.5026**	**0.5197**	**0.5315**	**0.5536**

Note: First, the evaluation score of *NTU* in each province in each year was calculated using CRITIC, then, the average score of each geographical region in each year was calculated based on them.

**Fig 2:**
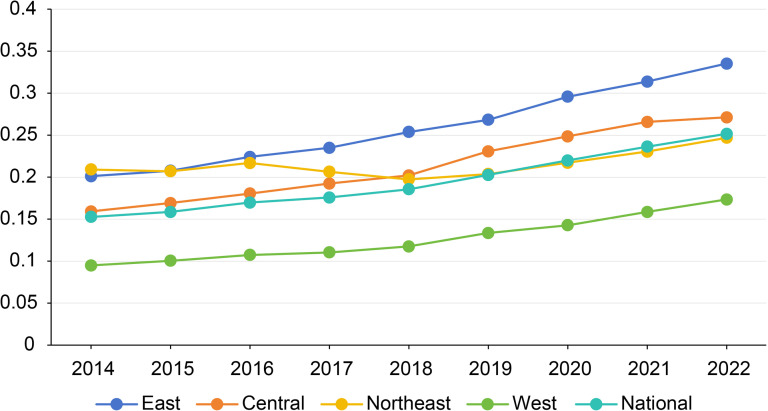
Trends of the rural digital economy level in the four geographical regions. Note: Compiled by the author based on the computed *Dige* evaluation score of each geographical region.

**Fig 3:**
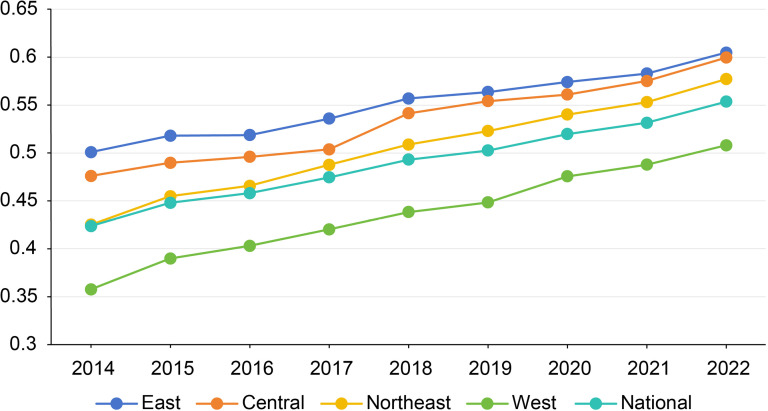
Trends of the new-type urbanization level in the four geographical regions. Note: Compiled by the author based on the computed *NTU* evaluation score of each geographical region.

The morphology of the curves in Fig 2 and 3 indicates that the changing trends of the two systems in the four geographical regions are basically in line with that of the whole nation, presenting a development pattern in graphic form as “East leading, Central trailing, Northeast and West catching up”. The regional disparities in the development of the rural digital economy and new-type urbanization is consistent with the economic development level of the four regions. Thanks to its favorable geographical location and preferential policy conditions, the eastern region has taken the lead in industrial agglomeration and talent attraction, thereby demonstrating the most prominent performance in these two systems. The central region trails closely behind the eastern region and has a relatively high growth rate. Due to the economic slowdown, the growth of various indicators of these two systems in the northeastern region has been sluggish and is lower than the national average level. The western region has been lagging behind the national level all the time, but it has begun to accelerate its development in recent years.

**Fig 4:**
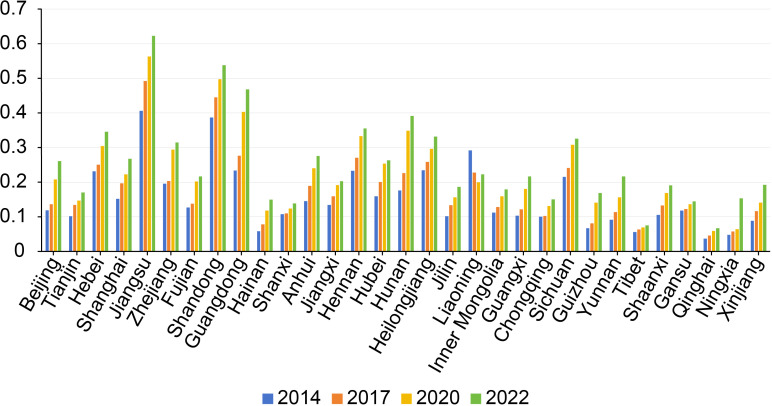
The rural digital economy level in the 31 provincial-level administrative regions. Note: Compiled by the author based on the calculated *Dige* evaluation score of each provincial-level administrative region.

**Fig 5:**
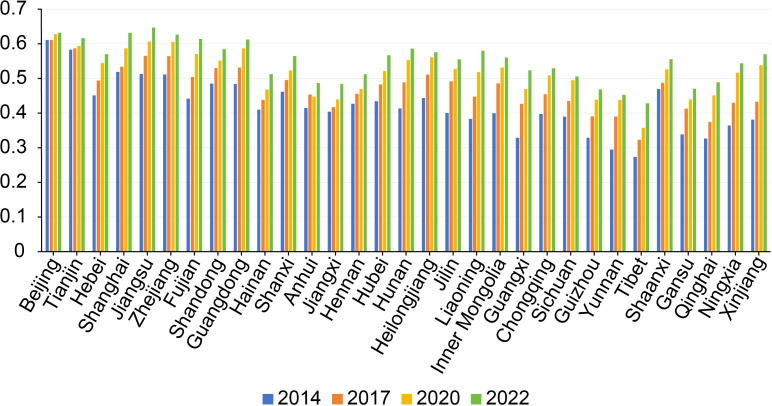
The new-type urbanization level in the 31 provincial-level administrative regions. Note: Compiled by the author based on the calculated *NTU* evaluation score of each provincial-level administrative region.

This study also selects four time nodes (2014, 2017, 2020, and 2022) to depict the evolution trend and regional disparity of the two systems. Based on the graphical depiction in Fig 4, the development level of the rural digital economy has been continuously rising during the research period, but the regional disparities among different provinces seem rather obvious. For example, Jiangsu, Shandong, and Guangdong maintain a relatively high growth trend and rank among the top. Some provinces in the central, northeast, and western regions, such as Hunan, Henan, Anhui, Heilongjiang, and Sichuan, exhibit strong growth momentum. In contrast, most provinces in the northeast and western regions are relatively underdeveloped yet harbor great potential. Fig 5 shows that new-type urbanization in central and western China is developing very fast despite starting late. The development pattern of new-type urbanization has evolved from an obvious spatial distribution of “High in the east, low in the west” to a balanced development pattern across the country, with inter-provincial gap still existing.

### Global-local spatial auto-correlation analysis

The above analysis is based on the graphical representation. To better examine the clustering and dispersion characteristics of the research objects in spatial distribution, this study also conducted a global-local auto-correlation analysis using Stata 18.

#### Global spatial auto-correlation (Global Moran’s I).

According to the global Moran’s I shown in Table 7 (Scatter plots are not presented due to space limitations), it is found that from 2014 to 2022, the spatial auto-correlation of the rural digital economy level has always been negative, and the p-values are all greater than 0.05, failing to reach statistical significance. The rural digital economy level does not exhibit significant spatial agglomeration or dispersion effects on a national scale, and its spatial distribution is relatively balanced. Although the rural digital economy level in some regions is relatively prominent, on the whole, the differences between provinces are not significant. The spatial auto- correlation of the rural digital economy level is weak, and there is no strong regional aggregation or difference.

**Table 7:** Global Moran’s I.

Global Moran’s I						
Year	I	p-value	z	I	p-value	z
	Dige			NTU		
2014	−0.145	0.205	−1.266	−0.209	0.052^*^	−1.945
2015	−0.156	0.156	−1.42	−0.207	0.054^*^	−1.925
2016	−0.149	0.183	−1.332	−0.197	0.072^*^	−1.798
2017	−0.162	0.136	−1.491	−0.205	0.058^*^	−1.896
2018	−0.162	0.135	−1.495	−0.182	0.1	−1.643
2019	−0.138	0.236	−1.185	−0.166	0.144	−1.463
2020	−0.129	0.281	−1.078	−0.134	0.267	−1.11
2021	−0.122	0.317	−1	−0.139	0.25	−1.151
2022	−0.089	0.529	−0.63	−0.113	0.384	−0.87

The spatial distribution of new-type urbanization level presents spatial dispersion effect, that is, within a certain spatial scope, there is a certain anti-correlation between the inter-regional new-type urbanization level. There are more low-*NTU* level regions around high-*NTU* level regions, which is so called “polarization” phenomenon. It is worth noting that, the new-type urbanization level from 2014 to 2017 shows some characteristics of negative spatial correlation, with great differences among regions. However, the Moran’s I and the significance level have been gradually decreasing since 2018, suggesting that the new-type urbanization level among regions tends to be balanced. This changing trend may be closely related to the gradual promotion of regional coordinated development policy and rural revitalization strategy.

#### Local spatial auto-correlation (Local Moran’s I).

As shown in Table 8, the p-values of most provinces exceed 0.05, including those of Beijing (0.787), Hebei (0.764), and Liaoning (0.712), etc. This indicates that the deviation of the Local Moran’s I of the rural digital economy in these regions from the expected values is insignificant, and the spatial auto-correlation is relatively weak. Notably, Shandong and Ningxia present highly statistically significant spatial auto-correlation. Such significantly negative values of Ii indicate that there is likely a phenomenon of low-value agglomeration, that is, the rural digital economy variables of the spatial units within these regions and those of the surrounding units show a state of low-level values clustering with each other. In some regions like Tianjin and Shanxi, the values of Ii are both positive and negative. Although these regions did not exhibit significant spatial auto-correlation in the statistical tests, they still deserve further attention as there may be certain spatial agglomeration or dispersion trends within local areas.

**Table 8:** Local Moran’s I.

Local Moran’s I (*Dige*)						Local Moran’s I (*NTU*)				
Province	Ii	E(Ii)	sd(Ii)	z	p−value^*^	Ii	E(Ii)	sd(Ii)	z	p−value^*^
Beijing	−0.111	−0.033	0.289	−0.27	0.787	−0.671	−0.033	0.293	−2.174	0.03
Tianjin	0.165	−0.033	0.228	0.868	0.385	−1.218	−0.033	0.23	−5.151	0
Hebei	−0.134	−0.033	0.334	−0.3	0.764	−0.073	−0.033	0.34	−0.116	0.907
Shanxi	0.13	−0.033	0.244	0.669	0.503	−0.025	−0.033	0.246	0.034	0.973
Inner Mongolia	−0.052	−0.033	0.184	−0.104	0.917	0.017	−0.033	0.184	0.276	0.783
Liaoning	−0.113	−0.033	0.215	−0.369	0.712	−0.077	−0.033	0.217	−0.203	0.839
Jilin	0.066	−0.033	0.2	0.495	0.62	0.001	−0.033	0.201	0.172	0.864
Heilongjiang	0.207	−0.033	0.561	0.428	0.669	−0.036	−0.033	0.573	−0.005	0.996
Shanghai	0	−0.033	0.306	0.111	0.912	−0.011	−0.033	0.311	0.071	0.943
Jiangsu	−0.179	−0.033	0.464	−0.315	0.753	−0.428	−0.033	0.473	−0.834	0.404
Zhejiang	0.038	−0.033	0.234	0.307	0.759	0.102	−0.033	0.236	0.572	0.567
Anhui	0.007	−0.033	0.297	0.134	0.893	−0.017	−0.033	0.301	0.054	0.957
Fujian	−0.112	−0.033	0.217	−0.362	0.718	0.009	−0.033	0.219	0.194	0.846
Jiangxi	−0.015	−0.033	0.147	0.127	0.899	0.044	−0.033	0.145	0.533	0.594
Shandong	−1.238	−0.033	0.338	−3.561	0	−0.251	−0.033	0.344	−0.634	0.526
Henan	−0.318	−0.033	0.553	−0.515	0.607	−0.174	−0.033	0.565	−0.249	0.803
Hubei	−0.036	−0.033	0.588	−0.005	0.996	0.025	−0.033	0.601	0.097	0.923
Hunan	0.021	−0.033	0.279	0.196	0.845	−0.009	−0.033	0.283	0.084	0.933
Guangdong	0.034	−0.033	0.291	0.231	0.818	−0.191	−0.033	0.296	−0.534	0.593
Guangxi	−0.769	−0.033	0.482	−1.527	0.127	−0.728	−0.033	0.492	−1.412	0.158
Hainan	−0.177	−0.033	0.602	−0.239	0.811	0.03	−0.033	0.615	0.104	0.918
Chongqing	−0.577	−0.033	0.592	−0.918	0.359	−0.124	−0.033	0.605	−0.15	0.881
Sichuan	0.089	−0.033	0.205	0.595	0.552	−0.078	−0.033	0.206	−0.216	0.829
Guizhou	0.129	−0.033	0.18	0.899	0.368	−0.218	−0.033	0.18	−1.025	0.305
Yunnan	−0.403	−0.033	0.561	−0.658	0.51	−0.68	−0.033	0.573	−1.128	0.259
Tibet	0.11	−0.033	0.204	0.701	0.483	−0.711	−0.033	0.205	−3.306	0.001
Shaanxi	−0.033	−0.033	0.181	0.004	0.997	−0.029	−0.033	0.181	0.026	0.979
Gansu	−0.167	−0.033	0.296	−0.45	0.652	−0.337	−0.033	0.3	−1.011	0.312
Qinghai	0.05	−0.033	0.229	0.365	0.715	−0.213	−0.033	0.231	−0.777	0.437
Ningxia	−1.034	−0.033	0.325	−3.077	0.002	−0.189	−0.033	0.33	−0.471	0.638
Xinjiang	−0.079	−0.033	0.327	−0.139	0.889	−0.228	−0.033	0.332	−0.587	0.557

The p-values of most provinces exceed 0.05, such as Hebei (0.907) and Shanxi (0.973), etc., indicating that the spatial auto-correlation of new-type urbanization in these regions is relatively weak and is closer to a random distribution state. However, the new-type urbanization in Beijing, Tianjin and Tibet displays significant spatial auto-correlation features. Since the Ii values in these regions are negative and the p-values are significant, it indicates that there is a pronounced negative spatial auto-correlation among them. That is to say, the new-type urbanization in these regions presents a distribution pattern where low values are adjacent to high values, which might be due to the differences in urban functional zoning.

#### Local spatial auto-correlation (Geary’s C).

As shown in Table 9, the p-values of most regions exceed 0.05, suggesting that the differences between the Geary’s C index in these regions and the expected 2.067 are insignificant, indicating the spatial *Dige* data distribution is near a normal or random state. A few regions show significant differences. For example, Jiangsu (with a p-value of 0.003) and Shandong (with a p-value of 0.000) have ci values far above the expected level, reaching a significant degree. This shows that the spatial *Dige* distribution in these regions differs significantly from expected values, possibly with relatively strong spatial agglomeration, dispersion, or other special spatial dependence relationships. It is worthwhile to conduct further research into the driving factors behind this, such as unique regional policies, geographical environment, industrial agglomeration, etc.

**Table 9:** Geary’s C index.

Geary’s ci (*Dige*)						Geary’s ci (*NTU*)				
Province	ci	E(ci)	sd(ci)	z	p−value^*^	ci	E(ci)	sd(ci)	z	p−value^*^
Beijing	1.434	2.067	2.007	−0.315	0.753	8.089	2.067	1.609	3.742	0
Tianjin	1.146	2.067	1.943	−0.474	0.636	8.173	2.067	1.543	3.958	0
Hebei	1.658	2.067	2.063	−0.198	0.843	0.654	2.067	1.667	−0.848	0.397
Shanxi	1.033	2.067	1.958	−0.528	0.597	1.108	2.067	1.559	−0.615	0.538
Inner Mongolia	1.316	2.067	1.905	−0.394	0.693	0.698	2.067	1.503	−0.911	0.362
Liaoning	3.404	2.067	1.931	0.692	0.489	1.95	2.067	1.531	−0.076	0.939
Jilin	1.114	2.067	1.918	−0.496	0.62	0.934	2.067	1.517	−0.746	0.455
Heilongjiang	2.452	2.067	2.427	0.159	0.874	0.946	2.067	2.032	−0.551	0.581
Shanghai	0.6	2.067	2.027	−0.723	0.469	2.182	2.067	1.63	0.071	0.943
Jiangsu	8.875	2.067	2.256	3.018	0.003	2.966	2.067	1.861	0.483	0.629
Zhejiang	0.785	2.067	1.948	−0.658	0.511	1.876	2.067	1.548	−0.123	0.902
Anhui	0.61	2.067	2.017	−0.722	0.47	0.865	2.067	1.619	−0.742	0.458
Fujian	1.727	2.067	1.933	−0.176	0.86	0.965	2.067	1.532	−0.719	0.472
Jiangxi	0.835	2.067	1.879	−0.656	0.512	1.16	2.067	1.476	−0.614	0.539
Shandong	10.251	2.067	2.068	3.957	0	1.897	2.067	1.672	−0.101	0.919
Henan	2.118	2.067	2.412	0.021	0.983	2.936	2.067	2.017	0.431	0.667
Hubei	1.072	2.067	2.478	−0.402	0.688	0.26	2.067	2.082	−0.868	0.386
Hunan	0.86	2.067	1.996	−0.604	0.546	0.732	2.067	1.598	−0.835	0.404
Guangdong	1.531	2.067	2.01	−0.266	0.79	1.893	2.067	1.612	−0.108	0.914
Guangxi	5.843	2.067	2.286	1.652	0.099	3.797	2.067	1.892	0.915	0.36
Hainan	1.768	2.067	2.504	−0.119	0.905	0.229	2.067	2.108	−0.872	0.383
Chongqing	3.469	2.067	2.486	0.564	0.573	1.005	2.067	2.09	−0.508	0.612
Sichuan	1.032	2.067	1.922	−0.538	0.59	1.282	2.067	1.521	−0.516	0.606
Guizhou	1.67	2.067	1.902	−0.208	0.835	3.641	2.067	1.5	1.05	0.294
Yunnan	2.168	2.067	2.428	0.042	0.967	4.969	2.067	2.033	1.428	0.153
Tibet	1.956	2.067	1.921	−0.058	0.954	6.733	2.067	1.52	3.07	0.002
Shaanxi	1.461	2.067	1.903	−0.318	0.75	1.578	2.067	1.501	−0.326	0.745
Gansu	1.402	2.067	2.015	−0.33	0.741	2.603	2.067	1.617	0.331	0.74
Qinghai	2.325	2.067	1.944	0.133	0.894	2.768	2.067	1.544	0.454	0.65
Ningxia	6.279	2.067	2.051	2.054	0.04	1.728	2.067	1.654	−0.205	0.838
Xinjiang	1.088	2.067	2.053	−0.476	0.634	1.633	2.067	1.657	−0.262	0.794

As for new-type urbanization, the p-value of most regions exceeds 0.05, indicating that the spatial distribution of new-type urbanization in these regions is closer to a normal or random distribution state, and the spatial auto-correlation is not highly pronounced. Notably, the ci values of Beijing (with a p-value of 0) and Tianjin (with a p-value of 0) are significantly higher than E(ci), being 8.089 and 8.173 respectively. This indicates that the spatial distribution of new-type urbanization in these two cities presents highly non-random agglomeration characteristics. This agglomeration may be associated with the strong attraction of urban core functional areas. For example, being the national capital, Beijing concentrates on a large number of administrative, cultural, commercial and other resources. Tianjin’s port economy, industrial bases may also facilitate the agglomeration of relevant factors.

### Variables descriptive statistics

The descriptive statistics results in Table 10 show that the development levels of both the rural digital economy and new-type urbanization exhibit evident differences in both temporal and spatial dimensions. The variance expansion factor (VIF) test indicates that the variables selected in this paper do not have severe multicollinearity issues and are suitable for the subsequent empirical analysis. Before the regression analysis, the model type should be selected as well. For panel data analysis, common models include fixed, random, and mixed effects models. This paper first examined the mixed-effects model using the F test. The results showed an F statistic of 34.09, a Prob value of 0.0000, and a 1% significance level. The null hypothesis of the mixed effects model is thus rejected, indicating that fixed or random effects models should be used. Subsequently, the Hausman test was used to further determine the model type, showing a chi-square statistic of 70.06 and a P-value of 0.0000 (less than 0.05), rejecting the null hypothesis of the random effect model. Therefore, the fixed-effects model is finally chosen for the analysis in this paper.

**Table 10:** Descriptive statistics of variables and multicollinearity test results.

Variable	Obs	Mean	Std. Dev.	Min	Max	Test results			
NTU	279	0.489	0.076	0.273	0.647	Multiple Collinearity Tests	Variable	VIF	1/VIF
Dige	279	0.195	0.113	0.037	0.623		Ope	3.91	0.255588
Inv	279	0.826	0.277	0.204	1.507		Tec	3.82	0.262108
Tec	279	1.761	1.164	0.189	6.53		Inv	2.32	0.431724
Ope	279	0.247	0.262	0.008	1.342		Pov	2.31	0.432828
Tra	279	0.473	0.477	0.052	3.282		Pop	2.12	0.472056
Ind	279	1.416	0.734	0.665	5.244		Ind	1.9	0.52765
Pov	279	0.079	0.009	0.051	0.094		Tra	1.79	0.55728
Pop	279	0.155	0.042	0.07	0.267		Dige	1.77	0.564194
Equ	279	0.43	0.048	0.345	0.521		Equ	1.04	0.963988
HC	279	0.086	0.038	0.035	0.274		Mean VIF	2.33	
ED	279	3.691	2.586	0.128	11.468	F test that all u_i=0	F (30, 239) = 34.09	Prob > F = 0.0000	
IS	279	0.591	0.176	0.024	0.919	Hausman test results	Chi-square test value	70.06	
Int	279	0.931	0.058	0.368	1.014		P-value	0	

### Benchmark regression results

Based on the established formulae, a two-way fixed-effect model is constructed, in which the rural digital economy (*Dige*) acts as the core explanatory variable and new-type urbanization (*NTU*) as the explained variable. In order to further explore the impact of the rural digital economy on new-type urbanization, this paper performs the regression analysis by adding control variables gradually. By observing the change of the impact of rural digital economy on new-type urbanization under different combinations of control variables, the robustness of the results can be validated. Table 11 presents the baseline regression results after the stepwise introduction of the control variables.

**Table 11:** Baseline regression results.

Variables	(1)	(2)	(3)	(4)	(5)	(6)	(7)	(8)
	NTU	NTU	NTU	NTU	NTU	NTU	NTU	NTU
Dige	0.140^***^	0.169^***^	0.116^**^	0.154^***^	0.208^***^	0.211^***^	0.188^***^	0.187^***^
	(2.952)	(3.328)	(2.172)	(2.940)	(3.833)	(3.960)	(3.563)	(3.521)
Inv		−0.014	−0.014	−0.021^**^	−0.025^***^	−0.026^***^	−0.021^**^	−0.021^**^
		(−1.563)	(−1.503)	(−2.383)	(−2.764)	(−2.955)	(−2.459)	(−2.457)
Tec			0.017^***^	0.024^***^	0.025^***^	0.023^***^	0.027^***^	0.027^***^
			(2.768)	(3.875)	(4.148)	(3.956)	(4.615)	(4.609)
Ope				0.088^***^	0.097^***^	0.082^***^	0.078^***^	0.079^***^
				(4.338)	(4.832)	(4.007)	(3.891)	(3.916)
Tra					0.050^***^	0.027	0.006	0.007
					(3.091)	(1.584)	(0.355)	(0.371)
Ind						−0.030^***^	−0.020^**^	−0.019^**^
						(−3.313)	(−2.147)	(−2.102)
Pov							−4.572^***^	−4.569^***^
							(−3.133)	(−3.126)
Pop							−0.127	−0.129
							(−1.357)	(−1.377)
Equ								−0.011
								(−0.514)
_cons	0.462^***^	0.468^***^	0.448^***^	0.413^***^	0.377^***^	0.437^***^	0.088	0.093
	(49.814)	(46.425)	(36.292)	(28.870)	(20.560)	(17.129)	(0.700)	(0.736)
N	279	279	279	279	279	279	279	279
R^2^	0.953	0.954	0.955	0.959	0.960	0.962	0.964	0.964
F	8.714	5.604	6.395	9.862	10.087	10.592	10.206	9.073
province	Yes	Yes	Yes	Yes	Yes	Yes	Yes	Yes
year	Yes	Yes	Yes	Yes	Yes	Yes	Yes	Yes

Note: t-values are shown in parentheses; ***, **, and * indicate significance at the 1%, 5%, and 10% levels, respectively.

In column (1), only the fixed effects of year and province are controlled without considering the control variables. In this case, the regression coefficient of the rural digital economy is 0.140, significant at the 1% significance level, indicating that the rural digital economy plays a significant positive role in promoting new-type urbanization. With the gradual addition of more control variables, the regression coefficients of the rural digital economy remain robustly and keep significant at the 1% significance level. The regression results in column (1)-(8) show that when all control variables are added to the model, the regression coefficient of the rural digital economy reaches 0.187, still significant. With the gradual introduction of control variables, the R^2^ value of the model increases from 0.953 to 0.964, indicating that the addition of control variables enhances the fitting ability of the model. The above results validate that the rural digital economy plays a significant role in promoting new-type urbanization, and this influence shows high robustness under different model frameworks.

The regression results also reveal different effects of control variables on new-type urbanization. In particular, Degree of openness (*ope*) shows a significant positive effect with a regression coefficient of 0.079 at the 1% significance level. This suggests that opening up can effectively promote new-type urbanization. The possible reason is that the opening policy facilitates the introduction of external knowledge and technology and enhances regional technological innovation capabilities, so as to promote economic growth and the urbanization process. Similarly, scientific and technological progress (*Tec*) also has a significant positive impact on new-type urbanization with a regression coefficient of 0.027 at the 1% significance level. As the primary productive force, science and technology can improve the quality and efficiency of regional economic development, thus playing a positive role in new-type urbanization. Furthermore, the regression coefficient of transportation infrastructure (*Tra*) is 0.007. Transportation infrastructure, as the core condition for the flow of production factors, accelerates the circulation of resources, personnel, and information, and provides important support for urbanization.

However, the impact of fixed asset investment (*Inv*) on new-type urbanization is negative with a regression coefficient of 0.021 at the significance level of 1%. A possible reason is that fixed asset investment tends to preferentially support asset-heavy industries, such as construction, power, and energy. Moreover, as investment in fixed assets increases, commercial lands encroach on residential lands, leading to rising urban housing prices, thus, it has brought an inhibiting effect on the development of new-type urbanization. The negative impact of industrial structure upgrading (*Ind*) on new-type urbanization is also apparent, suggesting that the excessive concentration of the tertiary industry may affect the efficiency of resource allocation to a certain extent, which is not conducive to the coordinated development of multiple industries in the new-type urbanization process. Particularly, rural poverty rate (*Pov*) shows a significant inhibitory effect on new-type urbanization with a regression coefficient of -4.569 at the 1% significance level, indicating that the decrease in the rural poverty rate has a significant promoting effect on new-type urbanization, since the reduction of the rural poor population can release more labor resources and activate market vitality, thereby accelerating the process of urban-rural integration. Simultaneously, the aging of the population and rural income inequality may also exert some negative influences on new-type urbanization.

### Addressing endogeneity issues

Table 12 shows the results of the endogenous regression. The sample consists of 30 provinces, excluding Tibet. In the first-stage regression analysis (column 1 and 3), the regression coefficients of the core instrumental variable (*IV*_*1*_) are both positive (0.044 and 0.0570, respectively) and significant at the 1% significance level. This finding indicates a significant positive correlation between the instrumental variable and the explanatory variable. The Wald F-values for the first stage are higher than the critical value of the weak instrumental variable, which further verifies the strong correlation of the instrumental variable. In the second-stage regression analysis (column 2 and 4), the regression coefficients of the rural digital economy with respect to new-type urbanization are 1.052 and 0.883, respectively, and are both significant at the 1% significance level. This outcome is in line with the benchmark regression result, further corroborating the significant positive impact of the rural digital economy on new-type urbanization. The regression results of the alternative instrument variable (*IV*_*2*_) also support the above conclusion (column 5–8).

**Table 12:** Results of endogenous test.

Variables	(1)	(2)	(3)	(4)	(5)	(6)	(7)	(8)
	Enogenicity test (*IV*_*1*_)				Enogenicity test (*IV*_*2*_)			
	Dige	NTU	Dige	NTU	Dige	NTU	Dige	NTU
IV	0.044^***^		0.057^***^		0.031		0.037^**^	
	(2.807)		(3.703)		(1.608)		(2.057)	
Dige		1.052^***^		0.883^***^		1.076^***^		0.600^***^
		(15.471)		(3.185)		(9.915)		(2.767)
Inv			0.032^***^	−0.043^***^			0.070^***^	−0.032^**^
			(2.898)	(−2.956)			(7.035)	(−2.572)
Tec			0.048^***^	−0.004			0.023^***^	0.005
			(5.930)	(−0.244)			(3.175)	(0.388)
Ope			−0.007	0.073^**^			−0.052^**^	0.050^*^
			(−0.236)	(2.474)			(−2.066)	(1.872)
Tra			0.014	0.137^***^			−0.320^***^	0.374^***^
			(0.675)	(6.279)			(−5.645)	(7.485)
Ind			0.021^*^	0.015			0.011	0.001
			(1.695)	(1.074)			(0.962)	(0.091)
Pov			−5.085^**^	0.416			2.943	−4.007
			(−2.444)	(0.144)			(1.513)	(−1.617)
Pop			0.377^***^	0.123			−0.363^***^	0.071
			(3.573)	(0.762)			(−3.201)	(0.532)
Equ			−0.019	0.004			−0.028	0.006
			(−0.576)	(0.130)			(−1.033)	(0.206)
_cons	−0.233	0.284^***^	−0.156	0.202	0.181^***^	0.280^***^	0.043	0.528^**^
	(−1.529)	(21.271)	(−0.584)	(0.828)	(16.108)	(12.897)	(0.269)	(2.534)
chi2	−	76398.235	−	112000		76147.68		149000
N	279	279	279	279	270	270	270	270
F	7.880	−	60.604	−	2.586		15.855	

Note: Data in parentheses are t-test values; ^***^, ^**^, ^*^ are significant at the 1%, 5%, and 10% levels, respectively.

In conclusion, the results of the endogeneity test demonstrate that the significant positive effect of the rural digital economy on new-type urbanization is robust. The results of the benchmark regression remain established after considering possible endogeneity.

### Robustness tests

Then, a robustness test is conducted to further verify the benchmark regression conclusion. The following robustness test methods were used in this study (Table 13): (1) Replacing calculation method. As presented in column (1), after altering the calculation method, the impact of the rural digital economy on new-type urbanization remains positive. The regression coefficient is 0.015, which is significant at the 1% level. Evidently, the positive impact of the rural digital economy on new-type urbanization is robust across different calculation methods. (2) Excluding special samples. As depicted in column (2), after excluding these special samples, the regression coefficient of the rural digital economy on new-type urbanization is 0.171, which is significant at the 5% level and is consistent with the benchmark regression results. Evidently, excluding some special samples does not alter the significantly positive impact of the rural digital economy on new-type urbanization. (3) Winsorization. The regression results presented in column (3) reveal that the regression coefficient of the rural digital economy is 0.193, significant at the 1% level. After the Winsorization treatment, the positive impact of the rural digital economy on new-type urbanization remains robust. This further excludes the possible interference of outliers on the model estimation. (4) Altering sample range. As presented in column (4), the regression coefficient of the rural digital economy is 0.148, significant at the 5% level. Even when the sample time range is shortened, the impact of the rural digital economy on new-type urbanization remains robust.

**Table 13:** Results of robustness test.

Variables	(1)	(2)	(3)	(4)
	Replacing calculation method	Excluding special samples	Winsorization	Altering sample range
	NTU	NTU	NTU	NTU
Dige	0.015^***^	0.171^**^	0.193^***^	0.148^**^
	(4.334)	(2.322)	(3.522)	(2.532)
Inv	−0.020^**^	−0.022^**^	−0.024^***^	−0.013
	(−2.409)	(−2.200)	(−2.696)	(−1.373)
Tec	0.025^***^	0.024^***^	0.027^***^	0.012^*^
	(4.265)	(3.699)	(4.596)	(1.833)
Ope	0.067^***^	0.070^***^	0.077^***^	−0.035
	(3.413)	(2.965)	(3.636)	(−0.970)
Tra	0.010	0.003	0.005	−0.004
	(0.540)	(0.144)	(0.287)	(−0.219)
Ind	−0.020^**^	−0.019^*^	−0.018^*^	−0.006
	(−2.260)	(−1.907)	(−1.858)	(−0.577)
Pov	4.577^***^	4.489^***^	4.029^***^	5.019^***^
	(3.172)	(2.817)	(2.690)	(3.246)
Pop	−0.135	−0.170	−0.120	−0.156
	(−1.469)	(−1.567)	(−1.265)	(−1.446)
Equ	−0.007	−0.013	−0.013	−0.050^**^
	(−0.311)	(−0.523)	(−0.605)	(−2.150)
_cons	0.133	0.118	0.136	0.138
	(1.075)	(0.849)	(1.058)	(1.054)
R^2^	0.965	0.961	0.960	0.968
F	9.984	6.686	8.350	4.984
N	279	243	272	217

Note: Data in parentheses are t-test values; ^***^, ^**^, ^*^ are significant at the 1%, 5%, and 10% levels, respectively.

The four robustness testing methods mentioned above all confirm that the rural digital economy has a significantly positive impact on new-type urbanization, and the benchmark regression result is robust. These findings further validate that the rural digital economy serves as a crucial driving force for new-type urbanization. Moreover, this conclusion is highly reliable and applicable across different scenarios and data-handling approaches.

### Structural analysis

The influence of the sub-dimensions of the rural digital economy on new-type urbanization. The rural digital economy is a multidimensional system, so it is necessary to consider the influence of its sub-dimensions on new-type urbanization. In this paper, the rural digital economy is divided into four sub-dimensions for regression analysis: rural digital infrastructure (*Dige-base*), digital transformation of agriculture (*Dige-agri*), agricultural production service informatization (*Dige-serv*) and digitization of rural life (*Dige-life*). The regression results in Table 14 show that the impacts of different dimensions of the rural digital economy on new-type urbanization are different.

**Table 14:** Structural regression results.

Variables		(1)	(2)	(3)	(4)	(5)	(6)	(7)	(8)	(9)
		Influence of the sub-dimensions of Dige				Influence on the sub-dimensions of NTU				
		NTU	NTU	NTU	NTU	Peo	Eco	Soc	Env	Coo
Dige	Dige-base	0.090***				0.407***	0.406***	0.070	0.129	-0.072
		(3.244)								
	Dige-agri		-0.015							
			(-0.489)							
	Dige-serv			0.150***		(3.029)	(4.077)	(0.532)	(1.129)	(-0.510)
				(3.413)						
	Dige-life				0.155***					
					(4.591)					
Inv		-0.012	-0.009	-0.017**	-0.022***	0.019	-0.037**	-0.065***	-0.042**	0.004
		(-1.543)	(-1.101)	(-2.023)	(-2.706)	(0.883)	(-2.282)	(-3.045)	(-2.260)	(0.159)
Tec		0.027***	0.033***	0.030***	0.029***	0.000	0.060***	0.082***	-0.009	0.038**
		(4.618)	(5.527)	(5.287)	(5.224)	(0.004)	(5.432)	(5.624)	(-0.676)	(2.433)
Ope		0.046**	0.061***	0.056***	0.064***	0.148***	-0.104***	0.147***	0.065	0.267***
		(2.205)	(2.630)	(2.781)	(3.294)	(2.900)	(-2.768)	(2.948)	(1.509)	(4.989)
Tra		-0.000	-0.017	-0.008	-0.008	-0.117**	0.070**	0.127***	0.043	-0.003
		(-0.024)	(-0.915)	(-0.443)	(-0.505)	(-2.543)	(2.057)	(2.818)	(1.095)	(-0.056)
Ind		-0.020**	-0.018*	-0.019**	-0.021**	-0.017	0.012	-0.056**	0.003	-0.035
		(-2.215)	(-1.909)	(-2.110)	(-2.358)	(-0.723)	(0.680)	(-2.465)	(0.145)	(-1.431)
Pov		4.168***	4.432***	4.196***	4.729***	12.525***	-1.802	4.041	3.727	-0.685
		(2.832)	(2.929)	(2.860)	(3.291)	(3.380)	(-0.657)	(1.112)	(1.187)	(-0.176)
Pop		-0.136	-0.199**	-0.192**	-0.191**	-0.342	-0.056	-0.280	0.674***	-0.926***
		(-1.444)	(-2.100)	(-2.088)	(-2.110)	(-1.440)	(-0.319)	(-1.202)	(3.346)	(-3.701)
Equ		-0.006	-0.015	-0.012	-0.005	0.031	0.002	-0.072	0.010	0.005
		(-0.290)	(-0.666)	(-0.547)	(-0.257)	(0.566)	(0.039)	(-1.331)	(0.206)	(0.089)
_cons		0.144	0.148	0.143	0.106	-0.533*	0.372	0.119	0.169	0.685**
		(1.142)	(1.129)	(1.141)	(0.855)	(-1.665)	(1.570)	(0.379)	(0.624)	(2.035)
N		279	279	279	279	279	279	279	279	279
R^2^		0.959	0.958	0.957	0.955	0.913	0.977	0.917	0.873	0.939
F		8.805	7.337	8.965	10.312	5.972	12.556	9.025	2.329	5.976

Note: Data in parentheses are t-test values; ^***^, ^**^, ^*^ are significant at the 1%, 5%, and 10% levels, respectively.

Column (1) show that the regression coefficient for the rural digital economy infrastructure (*Dige-base*) is 0.090, significant at the 1% significance level. This shows that the improvement of rural digital infrastructure can significantly promote the development of new-type urbanization. Good digital infrastructure provides technical support for the information circulation and resource sharing between rural and urban areas, while reducing the cost of urban-rural resource integration.

Column (2) show that the regression coefficient of digital transformation of agriculture (*Dige-agri*) is 0.155, significant at the 1% significance level, indicating the significant positive effect of digital agriculture on new-type urbanization. Digital agriculture greatly improves the efficiency and added value of agricultural economic activities, accelerates the development of rural economy, and further narrows the technological gap between urban and rural areas.

Column (3) show that the regression coefficient of agricultural production service informatization (*Dige-serv*) is 0.150, significant at the significance level of 1%, showing that agricultural production service informatization has a significant positive effect on new-type urbanization. By providing more precise technical support and market services for agriculture and rural industries, modern agricultural production service enhance agricultural production efficiency, inject new momentum into the rural economy, and expedite the flow of production factors between urban and rural areas.

Column (4) show that the regression coefficient of digitization of rural life (*Dige-life*) is -0.015, not significant. This indicates that digitization of rural life has no apparent facilitating effect effect on new-type urbanization. Compared with the other three dimensions, the current development level of *Dige-life* is relatively low, and there are substantial disparities among different regions. The developed eastern coastal areas are relatively mature in this regard, while the central and western regions are relatively backward. This might be the possible reason why *Dige-life* has not yet had a significant impact on new-type urbanization in the short term.

The influence of the rural digital economy on the sub-dimensions of new-type

urbanization. New-type urbanization encompasses five dimensions: population agglomeration (*Peo*), economic growth (*Eco*), social public service (*Soc*), environmental protection (*Env*) and urban-rural coordination (*Coo*). Building on the overall regression, this paper further incorporates these five dimensions as the explained variables into the model (see Table 13). The objective is to examine the impact of the rural digital economy on the diverse dimensions of new-type urbanization.

The regression results presented in columns (5) and (6) indicate that the rural digital economy has a significant and positive impact on population agglomeration (*Peo*) and economic growth (*Eco*), both being significant at the 1% significance level. This reveals that the rural digital economy has played a crucial role in facilitating population mobility, employment growth, and the improvement of income levels. As a vital component of new-type urbanization, population agglomeration (*Peo*) is closely associated with the development of the rural digital economy. The construction of digital infrastructure and the dissemination of information services promotes rural residents’ migration to cities. Meanwhile, the increase in employment opportunities and income further drives the development of economic growth (*Eco*).

The impact of the rural digital economy on social public service (*Soc*) is significant at the 10% level, and the impact is relatively weak, as shown in column (7). The possible reason is that although the digital economy has brought about profound changes to the residents’ social lifestyle through online education and smart transportation, these new business forms are mainly concentrated in urban areas. Therefore, the impact of the rural digital economy on social public service (*Soc*) has not been fully manifested.

Column (8) and (9) indicate that the impacts of the rural digital economy on environmental protection (*Env*) and urban-rural coordination (*Coo*) are not significant. These two dimensions are more influenced by the government’s ecological protection policies, industrial structure adjustment, economic development level, and other factors. The realization of environmental protection (*Env*) requires large-scale ecological restoration, pollution control, and green infrastructure construction, while urban-rural coordination (*Coo*) depends on the balanced allocation of urban and rural resources and policy support. At present, these areas have not yet exhibited the driving role of the rural digital economy.

### Spatial heterogeneity analysis

#### Geographical location.

Benchmark regression analysis is employed to explore the nationwide impact of the rural digital economy on new-type urbanization. However, due to the substantial differences in economic development levels, geographical conditions, and policy orientations among different regions in China, the development of the rural digital economy and new-type urbanization exhibits pronounced regional heterogeneous characteristics. Regression analyses have been conducted separately based on the panel data. The detailed results can be found in Table 15.

**Table 15:** Heterogeneity regression results.

Variables	(1)	(2)	(3)	(4)	(5)	(6)	(7)	(8)	(9)	(10)
	East	Central	West	Northeast	Lowpov	Highpov	Lowpop	Highpop	Lowequ	Highequ
	NTU	NTU	NTU	NTU	NTU	NTU	NTU	NTU	NTU	NTU
Dige	0.161	0.540^***^	0.031	0.390	0.192^***^	0.168	0.093	0.343^***^	0.249^***^	0.160^**^
	(1.528)	(3.907)	(0.233)	(1.058)	(3.360)	(1.412)	(0.858)	(5.549)	(2.909)	(2.183)
Inv	−0.012	0.035^**^	−0.003	0.062	0.007	−0.034^***^	0.000	−0.033^*^	−0.022	−0.004
	(−0.503)	(2.263)	(−0.228)	(1.418)	(0.717)	(−2.877)	(0.006)	(−1.861)	(−1.569)	(−0.360)
Tec	0.029^***^	−0.065^**^	−0.003	−0.014	0.004	0.042^***^	0.009	0.022^***^	0.030^***^	0.032^***^
	(3.690)	(−2.361)	(−0.204)	(−0.442)	(0.556)	(5.252)	(0.896)	(2.931)	(3.093)	(3.971)
Ope	0.028	0.003	−0.046	0.343^**^	0.078^***^	0.078	0.105^***^	−0.007	0.104^***^	0.080^***^
	(0.843)	(0.020)	(−0.761)	(2.412)	(3.471)	(1.130)	(3.479)	(−0.161)	(2.855)	(2.948)
Tra	0.002	0.162^*^	−0.041^*^	−2.027^**^	−0.038	−0.009	−0.040^*^	0.138^**^	−0.051	0.056^**^
	(0.010)	(1.936)	(−1.853)	(−3.255)	(−0.468)	(−0.379)	(−1.892)	(2.006)	(−1.021)	(2.328)
Ind	−0.031^*^	−0.052^***^	0.042^*^	0.042^**^	−0.008	−0.005	−0.024^*^	0.020	−0.002	−0.028^**^
	(−1.795)	(−2.777)	(1.941)	(2.592)	(−0.754)	(−0.254)	(−1.682)	(1.335)	(−0.121)	(−2.457)
Pov	4.525	−1.026	7.954^***^	0.438	0.984	−11.503^***^	6.209^**^	−1.619	10.599^***^	−0.636
	(1.493)	(−0.332)	(3.545)	(0.144)	(0.525)	(−3.515)	(2.324)	(−0.722)	(3.989)	(−0.320)
Pop	0.245^*^	−0.010	−0.551^***^	−0.309	−0.050	−0.379^**^	0.120	0.405^***^	−0.104	−0.021
	(1.761)	(−0.043)	(−2.673)	(−0.550)	(−0.460)	(−2.278)	(0.530)	(3.203)	(−0.618)	(−0.164)
Equ	0.061^*^	−0.006	−0.000	0.115	−0.027	−0.022	−0.018	0.028	0.049	−0.078
	(1.722)	(−0.197)	(−0.009)	(1.109)	(−1.063)	(−0.709)	(−0.585)	(1.068)	(0.653)	(−1.270)
_cons	0.086	0.541^**^	−0.166	1.013^**^	0.400^***^	−0.511^*^	−0.052	0.368^**^	−0.443^*^	0.492^***^
	(0.347)	(2.077)	(−0.777)	(3.000)	(2.870)	(−1.684)	(−0.213)	(2.189)	(−1.834)	(3.013)
N	90	54	108	27	140	135	138	139	139	138
R^2^	0.954	0.986	0.960	0.994	0.977	0.968	0.977	0.975	0.965	0.980
F	4.159	7.940	3.554	4.158	4.575	7.675	4.886	6.739	6.415	4.703

Note: Data in parentheses are t-test values; ^***^, ^**^, ^*^ are significant at the 1%, 5%, and 10% levels, respectively

The rural digital economy in eastern, central, and northeastern China has a positive impact on new-type urbanization, with certain differences in significance levels and influence coefficients. The regression coefficient of the central region is 0.540, which is significant at the 1% level, indicating that the rural digital economy has the strongest effect on new-type urbanization in the central region. However, the regression coefficients of the northeastern, eastern, and western regions are 0.390, 0.161, and 0.031, respectively, and are not significant.

The strong positive effect in the central region may be closely related to the “Rise of Central China” strategy since 2004. Policy support has introduced a vast array of digital resources and technologies, which inject vitality into rural economic development. In addition, the central region is in a stage of accelerated development, and the urgent need for economic growth and urban-rural integration further amplifies the driving effect of the rural digital economy.

Despite the strong policy support in Northeast China, the development of the rural digital economy has not been fully transformed into a significant driving force due to the long-term accumulated structural problems, such as industrial structure transformation and population outflow. Due to its long-standing leading position in the digital economy and urbanization, the rural digital economy in the east is relatively mature, and the driving force for growth is gradually weakening, which may explain its low coefficient and lack of significance.

The regression results in the west may be related to its relative disadvantage in terms of geographical environment, infrastructure, and resource allocation. The development level of the rural digital economy in the west is relatively backward, and the digital divide between urban and rural areas is more pronounced. However, with the increased investment in digital infrastructure in the west and the deepening of the rural revitalization strategy, the development potential of the rural digital economy in the west cannot be overlooked.

In addition, the regression results of the control variables also display variability among the regions. For instance, fixed asset investment (*Inv*) and transportation infrastructure (*Tra*) in the central region play a strong supporting role in new-type urbanization. Scientific and technological progress (*Tec*) has a significant positive impact on new-type urbanization in the east, which may be related to the regional differences in the structure of science and technology input and the utilization efficiency.

#### Rural poverty.

Rural poverty is introduced to further explore the spatial heterogeneity of the effect of rural digital economy on new-type urbanization. According to the mean value of rural poverty rate during the sample period, the samples are divided into low rural poverty rate group (*Lowpov*) and high rural poverty rate group (*Highpov*), and the regression results are shown in Table 15.

In the *Lowpov* group, the regression coefficient of the rural digital economy on new-type urbanization is 0.192, which is significant at the 1% level. This indicates that in areas with a low rural poverty rate, the rural digital economy plays a significant positive role in promoting new-type urbanization. This may be due to the high level of digital infrastructure and the digitization of production and daily life in areas with low rural poverty rate. In such areas, the digital economy is more likely to integrate into the local economic development and urbanization processes, thus having a strong positive impact. In contrast, in the *Highpov* group, the regression coefficient of the rural digital economy is 0.168 and is not significant. This indicates that in areas with high rural poverty rate, the rural digital economy plays a limited role in promoting new-type urbanization. This phenomenon may be related to the following factors: First, the digital infrastructure in areas with high rural poverty rate is relatively weak, and the development of the rural digital economy is still in its infancy, making it difficult to effectively promote new-type urbanization. Second, the level of economic development and human capital in high rural poverty rate areas is relatively low, so it is difficult to fully unleash the potential of the rural digital economy.

The regression results of the control variables also exhibit certain variability. For instance, in the *Lowpov* group, neither scientific and technological progress (*Tec*) nor degree of openness (*Ope*) exerts a significant impact on new-type urbanization. However, in the *Highpov* group, the regression coefficients of the two variables are 0.042 and 0.078, respectively, both being significant at the 1% level. This implies that the two variables are more crucial in areas with high rural poverty rate. Additionally, in the *Lowpov* group, the regression coefficient of fixed asset investment (*Inv*) is 0.007 and is not significant. In contrast, in the *Highpov* group, the regression coefficient is -0.034 and is significant at the 1% level. This indicates that in areas with high rural poverty rates, fixed asset investment (*Inv*) might be more concentrated in non-productive sectors, thereby inhibiting the urbanization process. Furthermore, population structure (*Pop*) in high rural poverty rate areas has demonstrated a significant negative impact on new-type urbanization. Higher proportion of aging population in high rural poverty rate areas may pose an obstacle to new-type urbanization.

It is worth noting that in the *Highpov* group, the regression coefficient of rural poverty (*Pov*) is -11.503 and is significant at the 1% level. This suggests that in areas with high rural poverty level, poverty may be a key factor influencing new-type urbanization. It further demonstrates the restrictive effect of high poverty rate on the development of the rural digital economy and new-type urbanization.

#### Population structure.

Population structure (elderly dependency ratio) is also introduced to further explore the spatial heterogeneity of this influence. According to the mean value of the elderly dependency ratio, the samples are divided into low elderly dependency ratio group (*Lowpop*) and high elderly dependency ratio group (*Highpop*), and regression analyses are conducted respectively.

The regression results in Table 15 indicate that in the *Highpop* group, the regression coefficient of the rural digital economy to new-type urbanization is 0.343, which is significant at the 1% level. Contrary to prior expectations, in regions characterized by a high elderly dependency ratio, the rural digital economy exerts a significantly positive impact on promoting new-type urbanization. In contrast, in the *Lowpop* group, the regression coefficient of the rural digital economy is 0.093 and is not significant. This indicates that in areas with a low elderly dependency ratio, the promotional effect of the rural digital economy on new-type urbanization is relatively weak. The aging level of a region is related to population inflow, fertility rate, job opportunities, and quality of life. Therefore, it is difficult to explore the influencing mechanism. For example, Jiangsu and Guangdong are both developed provinces along the southeastern coast of China. However, the relatively high level of aging in Jiangsu can be attributed to its comparatively long average life and low fertility rate. In contrast, the low aging level in Guangdong is mainly attributed to a large inflow of population and a high fertility rate resulting from the “Chaoshan” culture. A probable explanation is that areas with a high elderly dependency ratio typically face greater economic pressure and social security burdens, and the rural digital economy helps promote employment, boost income, and optimize resource allocation.

The regression results of the control variables further unveiled the disparities between the two sample groups. For instance, in the *Highpop* group, the regression coefficient of scientific and technological progress (*Tec*) on new-type urbanization is 0.022, significant at the 1% level. This indicates that technological innovation plays a particularly crucial role in promoting new-type urbanization in areas with a high elderly dependency ratio. Conversely, in the *Lowpop* group, the regression coefficient of scientific and technological progress (*Tec*) is only 0.009 and is not significant. This could be attributed to the fact that high dependency ratio areas rely on technological advancements to make up for labor shortages. In addition, the regression coefficient of degree of openness (*Ope*) in the *Lowpop* group is 0.105 and is significant at the 1% level. However, in the *Highpop*, the regression coefficient of degree of openness (*Ope*) is -0.007 and is not significant. This shows that in areas with an abundant labor force, openness can more effectively drive economic growth and the development of new-type urbanization.

#### Social inequality.

Social inequality is also incorporated for a spatial heterogeneity analysis. Based on the mean of the rural Gini coefficient, the samples are partitioned into the low social inequality group (*Lowequ*) and the high social inequality group (*Highequ*) for regression analysis.

The regression results in Table 15 indicate that, in the *Lowequ* group, the regression coefficient of the rural digital economy is 0.249, significant at the 1% level. This demonstrates that in regions with a relatively balanced income distribution, the rural digital economy has a more pronounced effect in promoting new-type urbanization. The reason is that the socio-economic environment in areas with low rural income inequality is more stable. Through efficient resource allocation and technology dissemination, the rural digital economy can have a more substantial impact on promoting economic growth and urbanization-related construction. However, in the *Highequ* group, the regression coefficient of the rural digital economy is 0.160 and is significant at the 5% level. This reveals that in areas with an uneven income distribution, the rural digital economy still exerts a certain promotional effect on new-type urbanization, yet the impact is relatively limited. This might be due to the low resource allocation efficiency in high social inequality areas. Additionally, the development of the rural digital economy fails to fully reach the low and middle income rural groups, thereby weakening its role in promoting new-type urbanization.

The regression results of the control variables also reflect the heterogeneity in income distribution differences. For instance, In the *Lowequ* group, scientific and technological progress (*Tec*) and degree of openness (*Ope*) play a particularly important role in new-type urbanization. In the *Highequ* group, the regression coefficients of these two variables are significant at the 1% level, but the impacts are relatively low. Moreover, transportation infrastructure (*Tra*) might assume a more significant role in areas with uneven income distribution, helping to bridge the urban-rural resource gap. Additionally, in the *Highequ* group, the regression coefficient of industrial structure upgrading (*Ind*) is -0.028, significant at the 5% level. This could be attributed to the fact that the tertiary industry may be less efficient in high income inequality areas, resulting in a weaker promotional effect on new-type urbanization.

### Mechanism analysis

In order to further explore the transmission mechanism of the rural digital economy impacting new-type urbanization, rural human capital improvement (*HC*), agricultural economy growth (*EG*), rural industrial structure upgrading (*IS*) and urban-rural market integration (*Int*) are introduced as the mediating variables for stepwise regression respectively, and the results are shown in Table 16.

**Table 16:** Validation of mediating effects.

Variables	(1)	(2)	(3)	(4)	(5)	(6)	(7)	(8)	(9)
	NTU	Mediating variables				NTU			
		HC	ED	IS	Int	HC	ED	IS	Int
Dige	0.187^***^	0.067^***^	8.739^***^	0.347^***^	0.047	0.112^**^	0.112^*^	0.132^***^	0.184^***^
	(3.521)	(3.880)	(7.075)	(2.699)	(0.503)	(2.190)	(1.947)	(2.647)	(3.478)
HC	—	—	—	—	—	1.116^***^	—	—	—
	—	—	—	—	—	(5.918)	—	—	—
ED	—	—	—	—	—	—	0.009^***^	—	—
	—	—	—	—	—	—	(3.089)	—	—
IS	—	—	—	—	—	—	—	0.158^***^	—
	—	—	—	—	—	—	—	(6.268)	—
Int	—	—	—	—	—	—	—	—	0.061
	—	—	—	—	—	—	—	—	(1.645)
Control	YES	YES	YES	YES	YES	YES	YES	YES	YES
_cons	0.093	0.099^**^	1.951	−0.286	0.945^***^	−0.018	0.076	0.138	0.035
	(0.736)	(2.414)	(0.664)	(−0.935)	(4.265)	(−0.150)	(0.613)	(1.178)	(0.266)
Province	YES	YES	YES	YES	YES	YES	YES	YES	YES
Year	YES	YES	YES	YES	YES	YES	YES	YES	YES
R^2^	0.964	0.985	0.983	0.960	0.809	0.969	0.966	0.970	0.965
Sobel test	Z					3.608	1.799	3.795	0.923
	P					0.000^***^	0.072^*^	0.000^***^	0.353
Bootstrap test	Indirect effect interval	—	—	—	—	0.0301131−0.0405456	0.000192− 0.00823	0.0080489−0.0331712	−0.0104766−0.0436609
	Direct effect interval	—	—	—	—	0.1286657−0.2313534	0.1670378−0.3918214	0.1286516−0.2223062	0.1206478−0.2299625
Contribution rate						39%	42%	29%	—

Note: Data in parentheses are t-test values; ^***^, ^**^, ^*^ are significant at the 1%, 5%, and 10% levels, respectively.

In column (1), a direct regression of the rural digital economy and new-type urbanization is conducted. The regression coefficient is 0.187 and is significant at the 1% level. This finding indicates that the rural digital economy significantly promotes new-type urbanization. Columns (2)-(5) directly regress the rural digital economy against each mediating variable. Specifically, the regression coefficients for rural human capital improvement (*HC*), agricultural economy growth (*EG*), and rural industrial structure upgrading (*IS*) are 0.067, 8.739, and 0.347 respectively. All of these coefficients are significant at the 1% level, which verifies the significant positive influence of the rural digital economy on the three mediating variables. However, the regression coefficient of urban-rural market integration (*Int*) is 0.047, and it fails the significance level test. This result demonstrates that the rural digital economy has a relatively weak impact on urban-rural market integration. Column (6)-(9) simultaneously conducts a regression on the rural digital economy, each mediating variable, and new-type urbanization. The coefficients of the rural digital economy are all positive and have passed the significance level test. The regression coefficients of rural human capital improvement (*HC*), agricultural economy growth (*EG*), and rural industrial structure upgrading (*IS*) are 1.116, 0.009, and 0.158, respectively, all being significant at the 1% level, with contribution rates of 39%, 42%, and 29%, respectively. Although the coefficient of urban-rural market integration (*Int*) is positive, the stepwise regression is not significant.

This paper used the Sobel test to verify the robustness of the mediating effect. The results demonstrate that the Z values of rural human capital improvement (*HC*), agricultural economy growth (*EG*), and rural industrial structure upgrading (*IS*) are 3.608, 1.799, and 3.795, respectively, while the P-values are 0.000, 0.072, and 0.000, respectively, which are significant at 1%, 10%, and 1% respectively. The Sobel test results once again validate the mediating roles of the three mediating variables, indicating a robust mediating effect. The Z value of urban-rural market integration is 0.923, which is not significant, indicating that the urban-rural market integration fails to act as a mediating variable.

This paper also used Sobel test to verify the robustness of the mediating effect. The results show that the Z value of rural human capital improvement (*HC*), agricultural economy growth (*EG*) and rural industrial structure upgrading (*IS*) are 3.608, 1.799 and 3.795, respectively, P-values are 0.000, 0.072 and 0.000, respectively, significant at 1%, 10% and 1% respectively. The Sobel test results once again verify the mediating roles of the three mediating variables, indicating a robust mediating effect. The Z value of urban-rural market integration is 0.923, not significant, indicating that the urban-rural market integration fails to serve as a mediating variable.

To further analyze the effect of the mediating variables, the original samples were sampled 1000 times using the Bootstrap method. The results show that the indirect and direct effects (with 95% confidence intervals) of rural human capital improvement (*HC*), agricultural economic growth (*EG*), and rural industrial structure upgrading (*IS*) are [0.0301131−0.0405456], [0.000192−0.00823], [0.0080489−0.0331712] and [0.1286657−0.2313534], [0.1670378−0.3918214], [0.1286516−0.2223062], respectively, excluding “0”, thus confirming the mediating roles of the three mediating variables between the rural digital economy and new−type urbanization. The indirect and direct effects (with 95% confidence intervals) of urban−rural market integration (*Int*) are [−0.0104766−0.0436609] and [0.1206478−0.2299625], respectively, including 0, indicating no mediating effect.

In summary, the three influence paths of “the rural digital economy—rural human capital improvement—new-type urbanization”, “the rural digital economy—agricultural economy growth—new-type urbanization”, and “the rural digital economy—rural industrial structure upgrading—new-type urbanization” exist significantly, thereby verifying the hypothesis 1 to hypothesis 3. However, the role of urban-rural markets integration is inconsistent with the theoretical expectation of hypothesis 4. This may be due to the fact that the urban-rural market integration is still in its nascent stage, and the efficiency of market factor flow and resource allocation still needs to be further enhanced. It has not yet formed a strong correlative effect between the rural digital economy and new-type urbanization.

## Discussion

The digital economy is becoming a new driving force for the future development of urbanization. Due to China’s long-existing dual economic structure, rural development lags far behind urban development, which has led to numerous problems in the urbanization process. Rural development should be an indispensable prerequisite for the healthy and sustainable development of urbanization. Against the backdrop of the Chinese government’s vigorous implementation of the rural revitalization strategy and the new-type urbanization strategy, it is even more necessary to conduct in-depth exploration of the mutually promoting relationship between the two. Therefore, it is of great value to explore how to enhance the driving force of rural development on new-type urbanization in the context of the rural digital economy. This study seeks to answer the critical question: can the rural digital economy effectively promote new-type urbanization? Our results are as follows.

First, this study has validated the substantial impact of the rural digital economy on new-type urbanization. Dualistic Economy Theory and Core-Edge Theory both underscore the economic radiation effect and the driving role of cities on rural areas [179]. Moreover, owing to the long-standing urban-rural dual economic structure, rural development in China has always lagged behind urban development [71,131]. Against this backdrop, the role of rural areas in urban development has always been neglected. Many problems that have emerged during the urbanization process, such as development imbalance and environmental damage, are closely associated with this situation [36,71]. Rural revitalization and new-type urbanization are two vital strategies that are closely intertwined and exert mutual impacts on each other. Existing research predominantly focuses on urban-related aspects. It mainly emphasizes the driving effect of the new-type urbanization strategy on the county-level economy and rural revitalization, yet it fails to adequately consider the counter-effect of rural development on new-type urbanization. This discovery expands the research perspective regarding the new-type urbanization issue and spurs more research efforts to explore practical approaches from the vantage point of rural development.

Second, structural analysis reveals that rural digital infrastructure, digital transformation of agriculture, and agricultural production service informatization have a significant impact on new-type urbanization at the current stage. In contrast, the role of rural life digitization is negligible. Digitization of rural life refers to the influence of digital-related industries on rural life, such as rural consumption, rural e-commerce, and rural finance. Owing to issues in infrastructure, digital literacy, and supply-demand matching, there are still numerous weaknesses and deficiencies in this area, and there are also obvious disparities among different regions. However, as digital-related industries expand in rural areas and balanced development is achieved among different regions, the potential impact of this indicator on new-type urbanization will further grow. Structural analysis also shows that at the present stage, the rural digital economy has a more significant positive impact on population agglomeration and economic growth, and has already exerted a certain influence on social public service. However, it has no significant impact on ecological environmental protection and the narrowing of urban-rural income and consumption gaps. As new business forms of the digital economy permeate into rural areas, its role in social public service, ecological protection and urban-rural coordination will be further strengthened.

Third, by conducting regression analyses on the sub-samples classified based on some important factors (such as geographical region, rural poverty, population structure, and social inequality), it is found that the regional heterogeneity of the impact is not only related to geographical regions but also manifested in the positive or negative impacts of the rural digital economy on new-type urbanization under different institutional frameworks and economic environments. However, existing research has primarily centered on the impact of urbanization in alleviating income inequality and reducing rural poverty [180–183], and scant research has been carried out regarding the implications of social inequality and rural poverty on urbanization, particularly in developing countries [184]. In this study, by incorporating relevant variables as control variables for benchmark regression and spatial heterogeneity analysis, the significant inhibitory effect of rural poverty on new-type urbanization was verified. Additionally, it was shown that the impact of the rural digital economy on new-type urbanization is constrained by rural poverty and rural inequality. Although this study has not uncovered the mechanism of aging, it does suggest that as the level of population aging in China gradually rises, the role of the rural digital economy in new-type urbanization will become more conspicuous. These results highlight the need for more targeted policies to boost the rural digital economy and new-type urbanization in line with geographical location, resource endowments, and institutional environments.

Finally, mediating mechanism analysis has identified agricultural economy growth, rural human capital improvement, and rural industrial structure upgrading as the transmission channels through which this impact is exerted. The insignificant impact of the rural digital economy on urban-rural market integration has hindered the verification of the mediating effect hypothesis. This is mainly because the rural digital economy is still in its initial stage and thus has difficulty in fully exerting its promoting effect on the integration of urban and rural market. At the industrial level, the digital transformation of traditional agriculture still lags behind and fails to precisely connect with the demands of urban industries, which hampers the coordinated progress of urban and rural commodity market. In addition, the inherent differences derived from the urban-rural dual structure have led to a significant gap in the consumption concepts between urban and rural residents, restricting the expansion of the consumption side of the rural digital economy and weakening its driving force for the integration of urban and rural consumption market. Selecting urban-rural market integration as a mediating variable is reasonable in theoretical logic, and it is not unexpected that it has failed to pass the mediating, mechanism verification. In China, where the urban-rural dual economic structure has been in place for a long time, the integration of urban-rural market is indeed a challenging task. Driven by the strong impetus of the digital economy, this effect is likely to be verified in the next few years.

While this study has achieved significant progress in understanding the subject, there are certain limitations and areas worthy of future exploration. One potential shortcoming of this study is that the selection of indexes is subjective and inevitably influenced by some personal bias. This is also an inherent limitation of the Comprehensive Evaluation Model. In addition, this study used a provincial-level panel data set from 2014 to 2022 for the empirical analysis. The starting year is 2014. In that year, the State Council of China held the Central Urbanization Work Conference for the first time. Subsequently, the “*National New-Type Urbanization Plan (2014—2020)*” was released, which indicated that urbanization in China had entered a stage of high-quality development. If the time span were longer, the research conclusions would be more convincing. It is worth noting that the evaluation index systems for the rural digital economy and new-type urbanization involve numerous indicators. For many of these indicators, county-level or municipal-level data are unavailable. Therefore, in this study, provincial-level data were adopted for the empirical analysis to ensure data availability.

Future studies should endeavor to overcome these limitations. The most important of these possibilities is that a combination of the Analytic Hierarchy Process (AHP) with the Entropy method or the Decision-Making Trial and Evaluation Laboratory (DEMATEL) model can assist in screening the evaluation indicators and reducing subjective bias in constructing the evaluation index system. With the gradual release of relevant statistical data, extending the panel data over a longer period of time will further enhance the accuracy of the empirical research. Additionally, future studies will attempt to utilize county and municipal level data to obtain more valuable conclusions. Moreover, more econometric models can be employed to expand the findings on this subject. For instance, the threshold effect model can be used to explore the nonlinear impact of the rural digital economy on new-type urbanization.

### Conclusion and policy implications

This study, based on the panel data of 31 provincial-level administrative regions in China (excluding Hong Kong, Macau, and Taiwan) from 2014 to 2022, verifies the impact and mechanism of the rural digital economy on new-type urbanization. The empirical results demonstrate that, during the study period, the rural digital economy has significantly propelled new-type urbanization. Currently, as the rural digital economy is in the nascent stage of development, its influence on new-type urbanization is gradually emerging. The impact of the rural digital economy on new-type urbanization varies substantially across different regions. In central China, which is in a stage of rapid development, the impact is particularly prominent. High levels of rural poverty and social inequality impose restrictive effects on this impact. Moreover, this impact is more pronounced in areas with a higher degree of aging. The rural digital economy promotes new-type urbanization through three mediating variables: rural human capital improvement, agricultural economic growth, and rural industrial structure upgrading. Among these factors, rural human capital improvement and agricultural economic growth contribute more significantly. The above-mentioned research findings not only clarify a novel practical path for China’s future new-type urbanization construction but also offer theoretical guidance and practical insights for policymakers in formulating appropriate support measures.

Based on the above research conclusion, the following countermeasures can be implemented. Firstly, enhance the rural digital economy from multiple perspectives to propel new-type urbanization. Given the negligible role of rural life digitization currently, it is necessary to explore certain strategies. For example, popularize digital payment systems and digital cultural and entertainment services in rural areas to reduce the digital divide between urban and rural regions. Secondly, actively promote the in-depth integration of digital technology into rural public services such as education and medical care. Develop digital-enabled ecological agriculture models to precisely manage pollution sources and strengthen environmental protection efforts. Moreover, make use of digital platforms to assist rural products in accessing broader markets, increase farmers’ income, and boost rural e-commerce. This can bridge the urban-rural income and consumption gaps, thereby contributing to the balanced development between urban and rural areas. Thirdly, based on the specific location conditions and resource endowments, formulate a dynamic and flexible development strategy for the rural digital economy. Investment in the rural digital economy should be appropriately inclined towards the western region. This is aimed at enhancing the breadth and depth of the coverage of the rural digital economy in the western region. Explore reasonable poverty alleviation projects based on local resources to improve the rural poverty situation, and take measures to narrow the rural income gap and mitigate the degree of rural income inequality. Given the growing aging of the population, offer more digital technology training to the rural elderly. This can assist them in adapting to the digital age. Simultaneously, encourage the innovation of rural digital economy models that are suitable for the elderly population. Finally, urban-rural market segmentation is the main obstacle in the urban-rural interaction mechanism. There is an urgent need to break down the market barriers between urban and rural areas, remove the obstacles to the free flow and equal exchange of production factors between them, and realize a virtuous circle of talents, lands, capital, industries, and information between urban and rural areas.
